# An immune gene signature to predict prognosis and immunotherapeutic response in lung adenocarcinoma

**DOI:** 10.1038/s41598-022-12301-6

**Published:** 2022-05-17

**Authors:** Hongquan Chen, Renxi Lin, Weibin Lin, Qing Chen, Dongjie Ye, Jing Li, Jinan Feng, Wenxiu Cheng, Mingfang Zhang, Yuanlin Qi

**Affiliations:** 1 School of Basic Medical Sciences, Fujian Medical University, Fuzhou, 350122 Fujian China; 2 Department of Pathology, Fujian Provincial Maternity Hospital, Fuzhou, 350012 Fujian China; 3 Luoyang Central Hospital Affiliated to Zhengzhou University, Luoyang, 471099 Henan China

**Keywords:** Immunology, Immune evasion, Immunotherapy, Tumour immunology, Cancer, Tumour biomarkers, Tumour immunology

## Abstract

Lung adenocarcinoma is one of the most common malignant tumors worldwide. The purpose of this study was to construct a stable immune gene signature for prediction of prognosis (IGSPP) and response to immune checkpoint inhibitors (ICIs) therapy in LUAD patients. Five genes were screened by weighted gene coexpression network analysis, Cox regression and LASSO regression analyses and were used to construct the IGSPP. The survival rate of the IGSPP low-risk group was higher than that of the IGSPP high-risk group. Multivariate Cox regression analysis showed that IGSPP could be used as an independent prognostic factor for the overall survival of LUAD patients. IGSPP genes were enriched in cell cycle pathways. IGSPP gene mutation rates were higher in the high-risk group. CD4 memory-activated T cells, M0 and M1 macrophages had higher infiltration abundance in the high-risk group, which was associated with poor overall survival. In contrast, the abundance of resting CD4 memory T cells, monocytes, resting dendritic cells and resting mast cells associated with a better prognosis was higher in the low-risk group. TIDE scores and the expressions of different immune checkpoints showed that patients in the high-risk IGSPP group benefited more from ICIs treatment. In short, an IGSPP of LUAD was constructed and characterized. It could be used to predict the prognosis and benefits of ICIs treatment in LUAD patients.

## Introduction

Lung cancer is the leading cause of cancer death worldwide. According to global tumor statistics in 2020, the fatality rate of lung cancer accounts for 18% of the total cancer deaths^1^. Lung cancer can be divided into small cell lung cancer (SCLC) and non-small-cell lung cancer (NSCLC). NSCLC accounts for approximately 85% of all lung cancers and can be divided into lung adenocarcinoma (LUAD) and lung squamous cell carcinoma (LUSC) based on its pathological characteristics^2^. LUAD is the most common type of non-small-cell lung cancer^3^. It is difficult to diagnose LUAD in the early stage, and thus it is usually diagnosed in the late stage (> 75%). Although the 5-year survival of patients with clinical stage IA is about 60%, the 5-year survival rate with clinical stage II-IV ranges from 40 to 5%^4,5^. Therefore, there is an urgent need for a new index to predict the survival rate and guide the clinical management of patients with lung adenocarcinoma.

In recent years, immunotherapy has become a hot spot in the treatment of lung adenocarcinoma^6^. Cancer immunotherapy overcomes tumor immune escape and reactivates the antitumor immune response by regulating the immune system^7^. Early methods of cancer immunotherapy targeted cytokines that affect the function of immune cells^8^, while the new generation of immune checkpoint inhibitors (ICIs) therapy targets immune checkpoint molecules, including cytotoxic T lymphocyte antigen-4 (CTLA-4), programmed cell death protein 1 (PD-1) and PD-1 ligand (PD-L1)^9^. ICIs had been used in NSCLC therapy^10^. For example, in an early-phase study involving patients with advanced NSCLC, the median duration of overall survival of patients was 17.1 months with combination of anti-CTLA-4 and anti-PD-1 antibodies and 13.9 months with chemotherapy^11^. However, compared with LUSC patients, LUAD patients have a lower response to ICIs therapy^11–13^. The effect of ICIs treatment can be modulated by many factors, including the tumor microenvironment (TME). Analysis of the TME is helpful to improve the effect of cancer immunotherapy and the prognosis^14^. Therefore, it is urgent to construct an immune gene marker to predict the efficacy and prognosis of LUAD immunotherapy.

Several prognostic biomarkers of LUAD have been established^15–18^. In this study, we developed a new immune gene marker that can predict the response to immunotherapy. By using weighted gene co-expression network analysis (WGCNA), immune hub genes related to LUAD patient prognosis and an immune gene signature for prediction of prognosis (IGSPP) were constructed. The relationships between IGSPP, the pathological features and the overall survival of LUAD patients were then studied. The molecular and immune characteristics of IGSPP were analyzed. The prognostic ability of IGSPP in patients with immunotherapy was detected and compared with other prediction models. We hope that this IGSPP can be used as a new prognostic biomarker for patients with LUAD.

## Methods

### Data collection

RNA-seq data and related clinical information of 535 LUAD samples and 59 para-cancer samples were downloaded from the TCGA database (https://portal.gdc.cancer.gov/). Samples with an unknown total survival time were excluded. The RNA-seq data and clinical information of 1134 LUAD patient samples (dataset GSE68465, GSE30219, GSE72094) were downloaded from the GEO database (https://www.ncbi.nlm.nih.gov/geo/). Immune-related gene (IRG) lists were downloaded from the ImmPort (https://www.immport.org/home) and InnateBD (https://www.innatedb.ca/) databases.

### Screening of immune hub genes

Gene expression data of 535 TCGA LUAD samples were extracted by Strawberry Perl (version 5.32.1.1). Differentially expressed genes (DEGs, |log_2_FC| > 1, q-value < 0.05) between tumors and para-cancer tissues were screened by using the R package "limma". A list of differentially expressed Immune-related gene (DEIRGs) was then obtained by intersecting the DEG and IRG lists.

WGCNA can be used to find clusters of highly related genes. We used WGCNA to find immune gene sets related to the LUAD patient prognosis and then constructed a similarity matrix based on our DEIRG expression data. The scale independence and average connectivity of the networks were tested with different power values (1–10). The appropriate power value was determined when the independent scale was greater than 0.85 and the connectivity was high. Then, the similarity matrix was transformed into a topological matrix with the topological overlap measure (TOM) describing the correlation between genes. The genes were clustered by using 1-TOM as the distance. The dynamic pruning tree was built, and the merging threshold function was set to 0.25 for module identification. Finally, the module with the highest correlation with the clinical features was selected as the key module. This module was used to identify immune-related hub genes^19^.

### Construction of IGSPP

We used the TCGA dataset as a training cohort and three GEO dataset (GSE68465, GSE30219, GSE72094) as validation cohorts (Table 1). The TCGA cohort was used to identify prognostic immune genes and to establish a prognostic-immune risk model. Three GEO cohorts were used to validate the predictive ability of the risk model. We used the R package "survival" to analyze hub genes in the TCGA cohort by using univariate Cox regression and then screened candidate genes significantly related to overall survival (OS). We used Kaplan–Meier (KM) plots to visualize the survival curves. Candidate genes were then integrated into least absolute shrinkage and selection operator (LASSO) regression. To prevent overfitting of the model, the R package "glmnet" was used to find the best gene model. Multivariate Cox regression analysis was then carried out, and the R package "survival" was used to identify genes significantly affecting OS. These genes were used to construct IGSPP. The risk scores of each sample in the TCGA and GEO cohorts were calculated according to the Cox regression coefficients and gene expression^20^.

**Table 1:** Clinical characteristics of patients with LUAD in each data set.

Characteristics	Training cohort	Testing cohort		
	TCGA	GSE68465	GSE30219	GSE72094
n	522	443	293	398
**State**				
Alive	334	207	93	285
Dead	188	236	200	113
**Age**				
≤ 65	241	231	179	118
> 65	262	212	113	280
Unknown	19	0	1	0
**Gender**				
Female	280	220	43	222
Male	242	223	250	176
**Stage**				
Stage I	279	0	0	254
Stage II	124	0	0	67
Stage III	85	0	0	57
Stage IV	26	0	0	15
Unknown	8	443	293	5
**T**				
T1	172	150	166	0
T2	281	251	69	0
T3	47	28	31	0
T4	19	12	21	0
Unknown	3	2	6	398
**N (%)**				
N0	335	299	198	0
N1	98	88	53	0
N2	75	53	30	0
N3	2	0	10	0
Unknown	12	3	2	398
**M (%)**				
M0	353	0	282	0
M1	25	0	8	0
Unknown	144	443	3	398

According to the median risk score, the samples were divided into high-risk groups (TCGA cohort, n = 252; GSE68465 cohort, n = 209; GSE30219 cohort, n = 200; GSE72094 cohort, n = 190) and low-risk groups (TCGA cohort, n = 252; GSE68465 cohort, n = 233; GSE30219 cohort, n = 93; GSE72094 cohort, n = 208). KM survival curves were used to compare the OS between subgroups. Time-dependent ROC curves were drawn by using the R package "timeROC" according to the risk scores. The area under the curve (AUC) was calculated to verify the accuracy and prediction ability of the model. Univariate and multivariate Cox regression analyses were performed to verify the independent prognostic value of IGSPP and explore the potential clinicopathological factors related to the prognosis. A nomogram was constructed by the R package "rms", and the predictive variables in multivariate analysis were used. Calibration curves were used to evaluate the consistency between the predictions and the actual survival results.

### Enrichment analysis

Gene ontology (GO) and Kyoto Encyclopedia of Genes and Genomes (KEGG) were analyzed by using the R package "clusterProfiler" (filtering condition: q-value < 0.05) to study the possible molecular mechanism of DEIRGs. The GO terms included biological process (BP), molecular function (MF) and cellular component (CC)^21^. The enrichment results were visualized with the R package "ggplot2".

Gene set enrichment analysis (GSEA) was used to analyze the enrichment differences of the functionally annotated gene sets according to the expression data. To explore the molecular mechanism of the difference in prognosis between the IGSPP subgroups, we used the R packages "limma" and "clusterProfiler" to analyze the GSEA enrichment in the hallmark gene sets (h.all.v7.4.symbols.gmt) and the KEGG subsets of the canonical pathway gene sets (c2.cp.kegg.v7.4.symbols.gmt).

### Tumor mutation burden assay

Mutation data of LUAD patients were downloaded from the TCGA portal (https://portal.gdc.cancer.gov/). Tumor mutation burden (TMB) was calculated by Strawberry Perl. Mutations of all 30 DEIRGs related to OS were visualized by the R package "maftools". Gene mutation data of the IGSPP subgroups were also extracted by Perl. Ten genes with the highest mutation frequency in the IGSPP subgroups were then visualized as waterfall charts by the R package "maftools". The correlations between TMB and the IGSPP risk score were analyzed. KM survival curves were used to show the effects of TMB on the survival rate on the IGSPP subgroups.

### Tumor immune microenvironment analysis

We used the CIBERSORT algorithm to estimate the immune cell infiltration^22^. According to the expression matrix of the TCGA cohort, the infiltration of 22 kinds of immune cells in 535 samples of LUAD patients was estimated. The relative ratios of the 22 types of immune cells between IGSPP subgroups were compared.

To further determine the immune and molecular characteristics between IGSPP subgroups, single sample GESA (ssGSEA) was performed on certain gene signatures, and the scores between two IGSPP subgroups were compared by using the R package "GSVA"^23^.

### Analysis of the prognostic value of IGSPP after ICIs therapy

Tumor immune dysfunction and exclusion (TIDE) score files of LUAD patients were downloaded from the TIDE website (http://tide.dfci.harvard.edu). The R package "limma" was used to analyze the differences among the IGSPP subgroups. We calculated the tumor inflammation signature (TIS) score of the LUAD sample of the TCGA cohort. Time ROC curves were drawn according to the IGSPP risk score, TIDE and TIS score by using the R package "timeROC" to verify the accuracy of the prediction by our IGSPP model.

### Cell lines and reagents

Human lung cancer cell lines (A549, H1299, PC9, H292, H1975) and human bronchial epithelial cell lines (Beas-2B) were purchased from the National Collection of Authenticated Cell Cultures (https://www.cellbank.org.cn, Shanghai, China). Cells were cultured in RPMI-1640 or F-12K medium (HyClone, Beijing, China) containing 10% fetal bovine serum (Ausbian, Australia) at 37 °C and 5% CO_2_, according to manufacturer’s instructions.

### RNA extraction and real-time quantitative PCR (RT-qPCR)

Total RNA were extracted from cultured cells by TRIzol (Invitrogen, Shanghai, China). Reverse transcription reactions were then performed by using a first-strand cDNA synthesis kit (novoprotein, Shanghai, China). Real-time PCR system was configured according to an ABI SYBR Green Master Mix (Applied Biosystems, USA), and the mRNA expressions of IGSPP genes were detected by using a real-time fluorescent quantitative PCR instrument (QuantStudio 3, Thermo Fisher Scientific, USA). 2^−ΔΔCt^ method was used to caculated the relative expression levels of the IGSPP candidate genes^24^. GAPDH was used as an internal reference. Primers used in RT-qPCR were listed in Table S1.

### Statistical analysis

Statistical analyses were carried out with R 4.1.0 (https://www.R-project.org) and GraphPad Prism 8.0 (GraphPad Software, San Diego, CA, USA). The differences in continuous variables between the two groups were tested by independent t-tests or nonparametric Wilcoxon tests. Categorical data were tested by chi-square tests. Univariate and multivariate Cox regression analyses were used to evaluate the effects of immune and clinicopathological factors on the prognosis of patients with LUAD. The Kaplan–Meier method and log-rank test were used for survival analysis. A two-sided p value < 0.05 was considered significant.

## Results

### Screening and characteristics of immune-related hub genes

**Figure 1:**
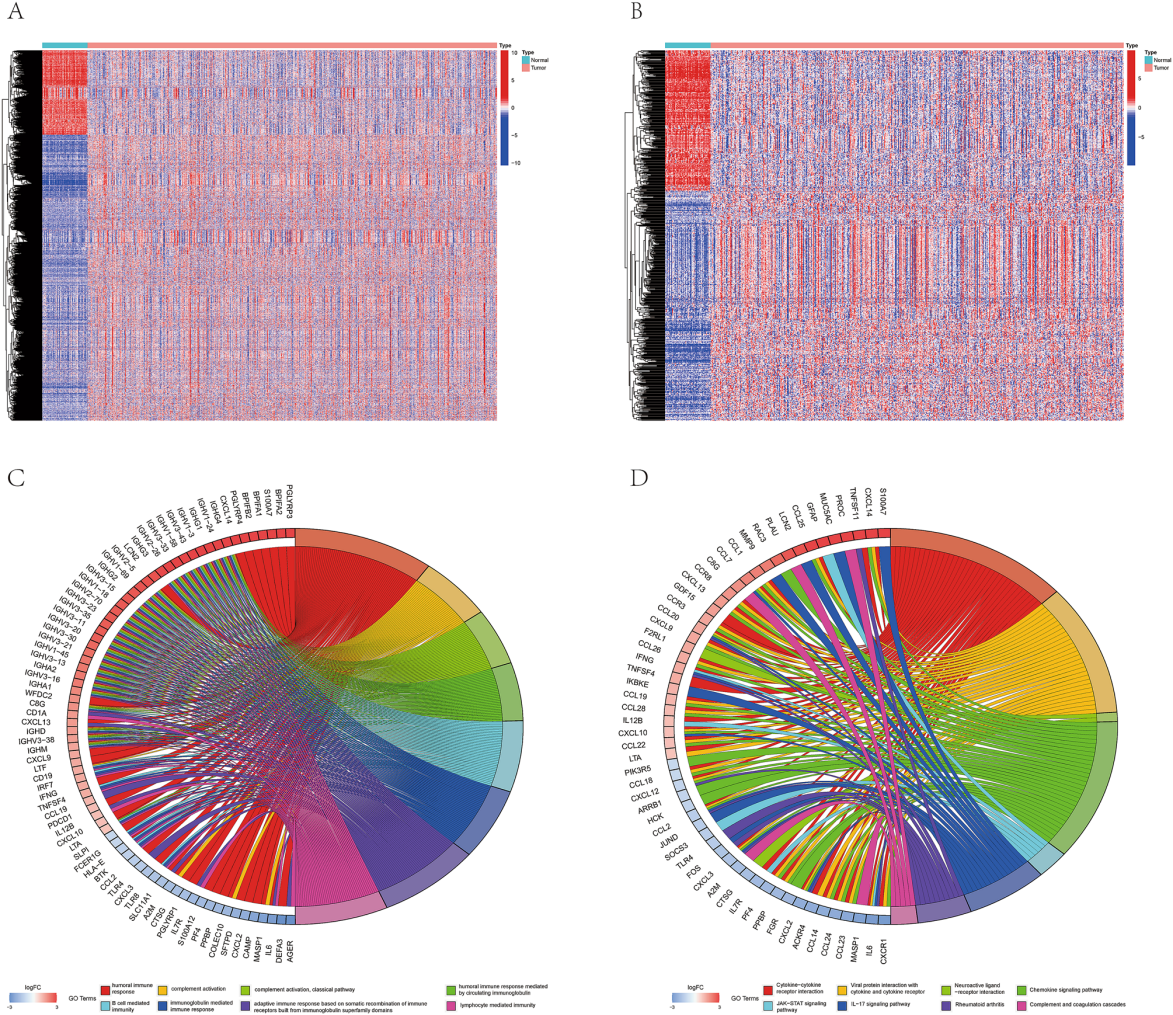
Differentially expressed immune-related genes in LUAD. (**A**) Differentially expressed genes (DEGs) (logFC filter = 1, FDR Filter = 0.05) between 535 LUAD samples (red) and 59 para-cancerous samples (blue). (**B**) Differentially expressed immune-related genes (DEIRGs) between 539 LUAD samples (red) and 59 para-cancerous samples (blue). (**C**) Gene Ontology (GO) enrichment analysis of DEIRGs (p < 0.05). (**D**) Kyoto Encyclopedia of Gene and Genome (KEGG) pathway analysis of DEIRGs (p < 0.05).

RNA sequencing data of 535 lung adenocarcinoma tissues and 59 normal tissues from the TCGA database were analyzed, and a total of 8191 differentially expressed genes (DEGs) (Table S2) were obtained. Among them, 6317 genes were upregulated and 1874 genes were downregulated (Fig. 1A). By overlapping DEGs and immune-related genes (IRGs) from ImmPort and InnateDB, 681 differentially expressed immune-related genes (DEIRGs) were obtained (Table S3). There were 258 downregulated and 423 upregulated genes among them (Fig. 1B).

GO analysis showed that the DEIRGs were enriched in humoral immune response, complement activation, B cell-mediated immunity, immunoglobulin-mediated immune response, and adaptive immune response based on somatic recombination of immune receptors built from immunoglobulin superfamily domains (Fig. 1C, Table S4). KEGG analysis^25–27^ showed that the DEIRGs were enriched in cytokine-cytokine receptor interaction, viral protein interaction with cytokines and cytokine receptors, neuroactive ligand-receptor interaction, chemokine signal pathway, JAK-STAT signal pathway, IL-17 signal pathway, rheumatoid arthritis and complement and coagulation cascade (Fig. 1D, Table S5).

**Figure 2:**
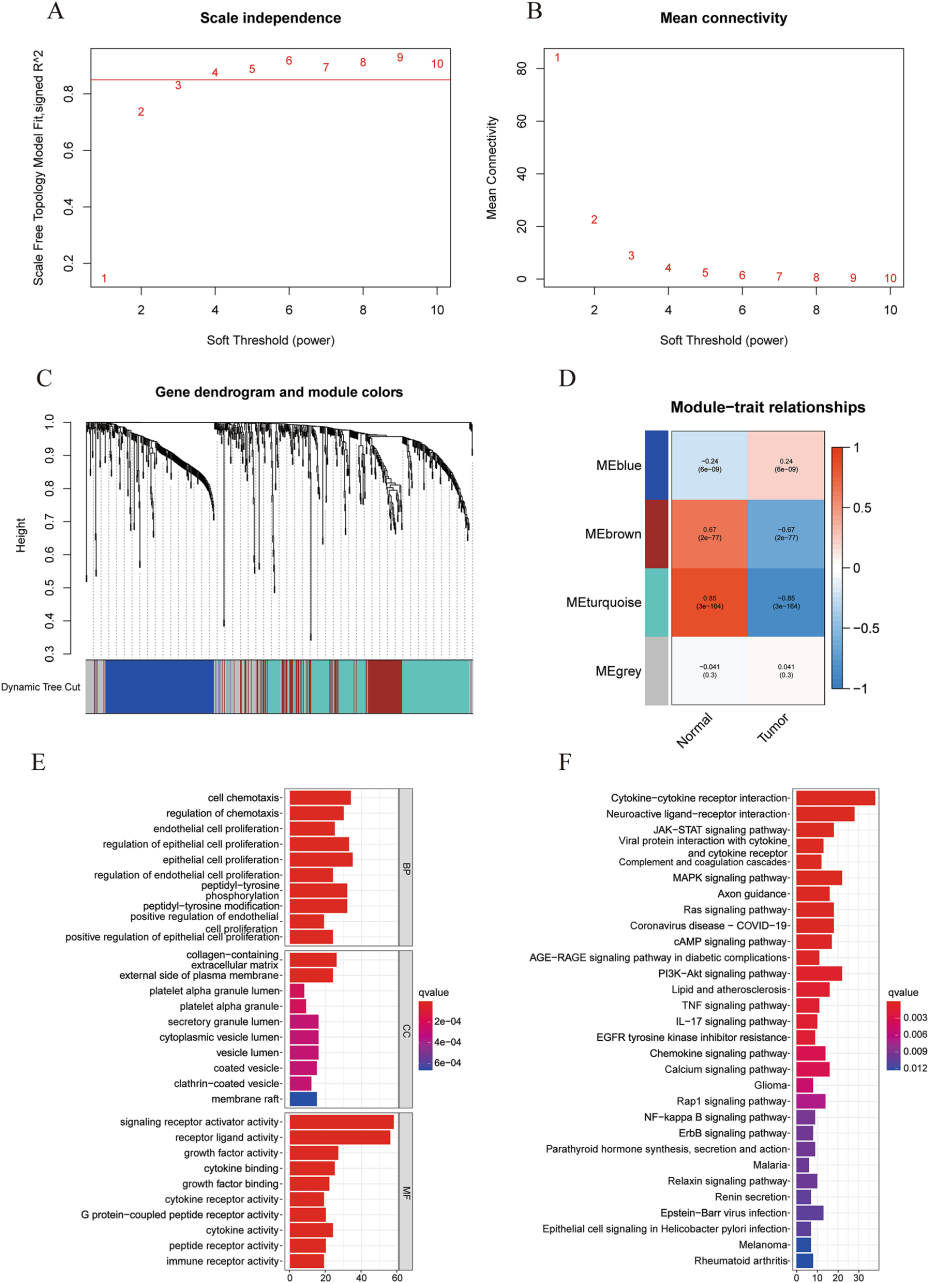
Identification of immune-related hub genes. (**A**) The horizontal threshold is 0.85. (**B**) The best soft threshold for WGCNA is 4. (**C**) DEIRG clustering tree based on dissimilarity measure (1-TOM). (**D**) The gene modules related to LUAD obtained by WGCNA. (**E**) Functional enrichment analysis of 263 genes in the turquoise module. (**F**) KEGG pathway enrichment analysis of 263 genes in the turquoise module.

To identify the module most related to prognosis, we performed WGCNA on the obtained DEIRGs. The optimal soft-thresholding power was 4 based on the scale-free network (Fig. 2A,B). Four modules were determined according to the average linkage hierarchical clustering and the optimal soft-thresholding power. The DEIRGs obtained above were allocated to four modules. According to the Pearson correlation coefficient between the module and the sample characteristics of each module, the turquoise module was found to be closely related to the prognosis of LUAD tumors (Fig. 2C,D). Therefore, we identified the turquoise module as the key module. A total of 263 genes in the turquoise module were selected for GO and KEGG analysis. Based on the p value, the top 10 enriched GO terms of BP, MF and CC (Fig. 2E) and the top 30 KEGG pathways are shown (Fig. 2F). For more information, please see Tables S6 and Table S7.

**Figure 3:**
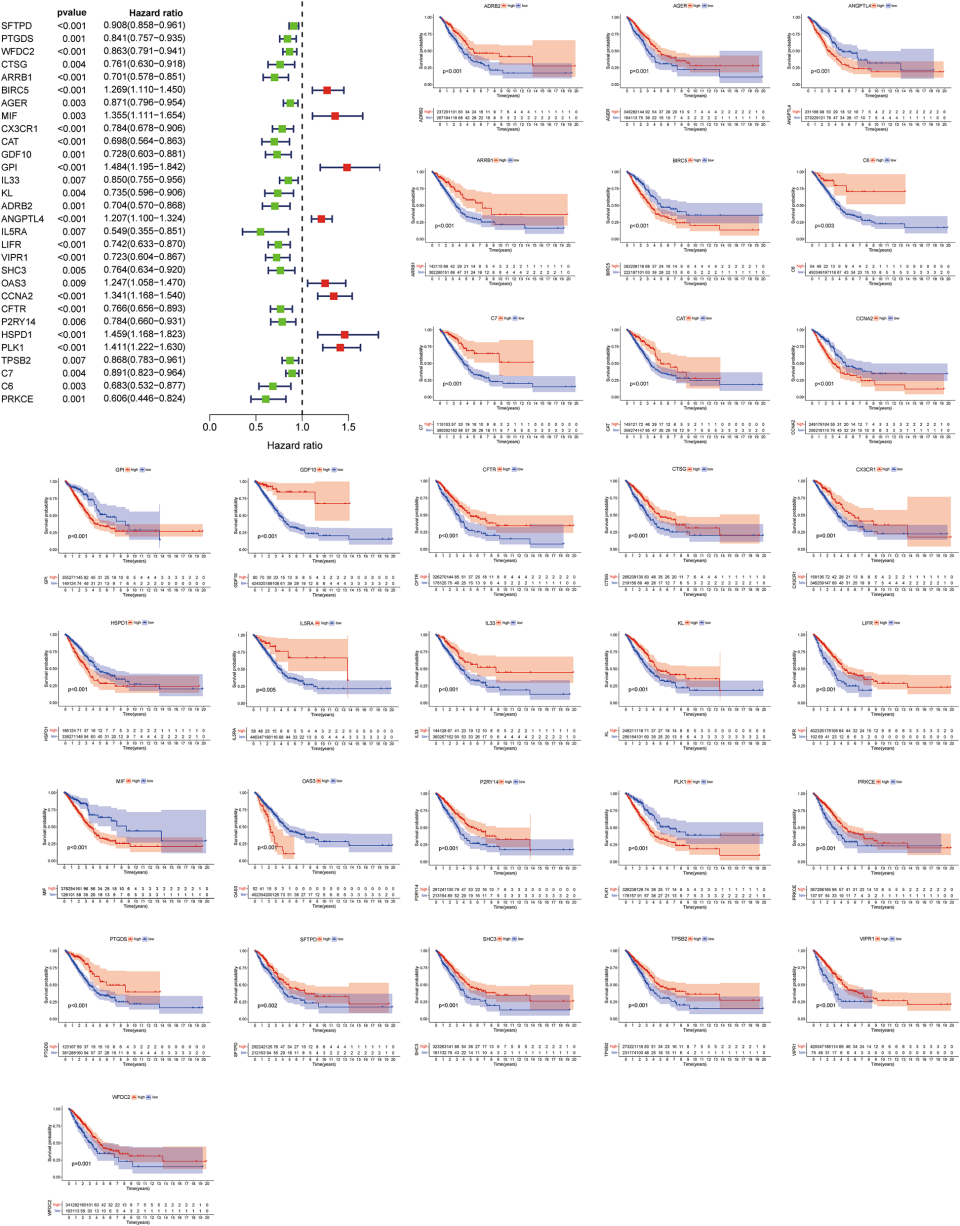
Kaplan–Meier survival curves of 30 immune-related hub genes obtained by univariate Cox regression analysis.

**Figure 4:**
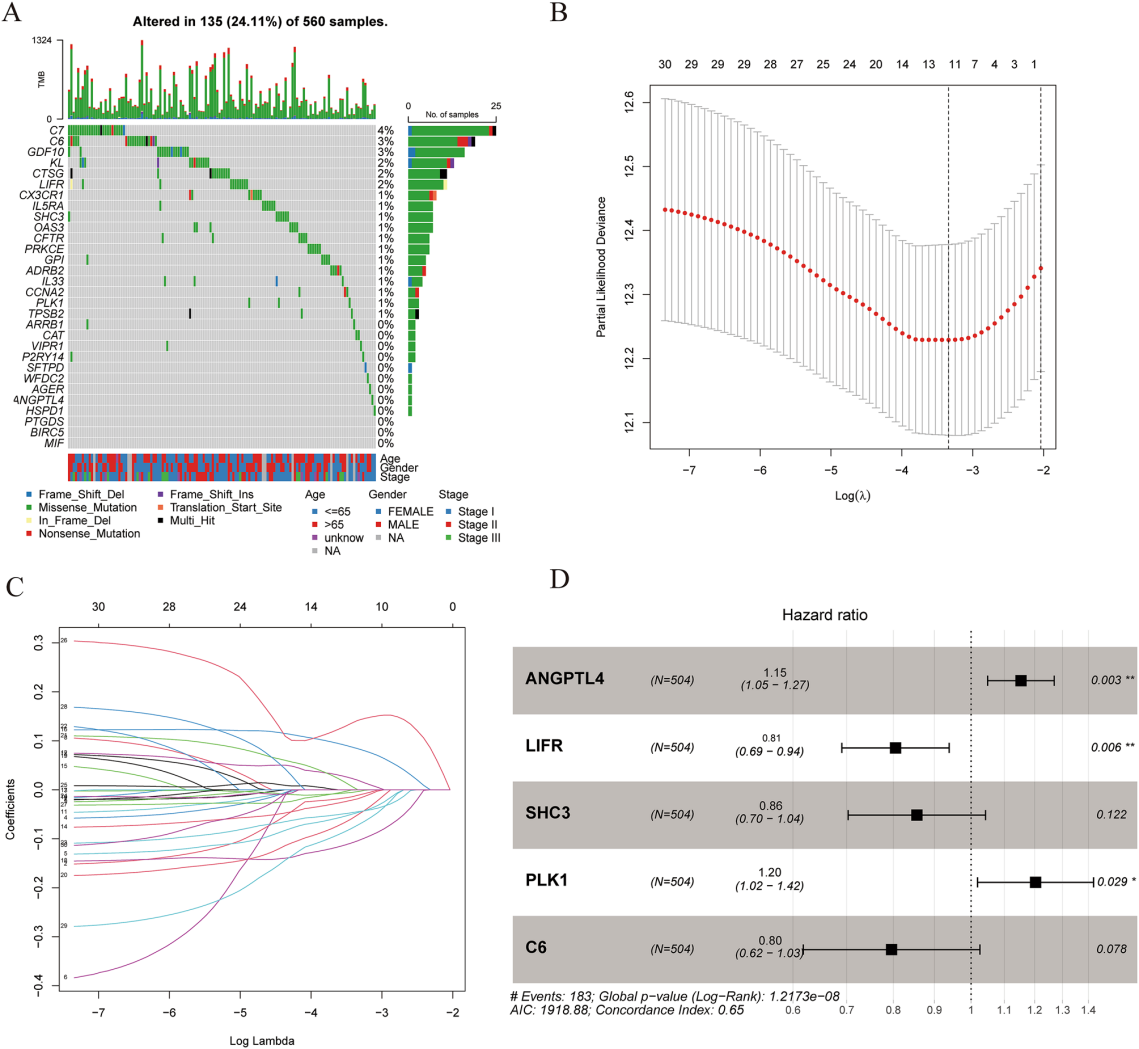
Construction of IGSPP for LUAD patients. (**A**) Mutation analysis of 30 immune-related hub genes. (**B**) The confidence interval of each λ. (**C**) Establishment of the LASSO regression model. (**D**) Multivariate Cox regression analysis was used to identify 5 immune-related hub genes used to construct the IGSPP.

The expression of turquoise module genes was extracted from the TCGA, GSE68465, GSE30219, GSE72094 datasets. A total of 210 intersecting genes were obtained. With a p value < 0.01 as the filtering condition, 30 DEIRGs related to OS were screened by univariate Cox analysis in the TCGA cohort (Fig. 3). We also analyzed the mutations of these 30 DEIRGs. Most of these genes had missense mutations, but the mutation rate was no more than 5% (Fig. 4A). To determine the independent prognostic genes, LASSO regression and multivariate Cox regression analyses were performed with the 30 DEIRGs obtained above (Fig. 4B,C). As shown in Fig. 4D, five genes (ANGPTL4, LIFR, SHC3, PLK1 and C6) were obtained and used to construct a prognostic index. IGSPP was constructed by multiplying the expression level of these genes by the Cox regression coefficient. The formula was IGSPP = expression level of ANGPTL4 × 0.14 + expression level of LIFR × (− 0.22) + expression level of SHC3 × (− 0.16) + expression level of PLK1 × 0.19 + expression level of C6 × (− 0.23).

### mRNA expressions of five IGSPP genes in cultured lung carcinoma cells

**Figure 5:**
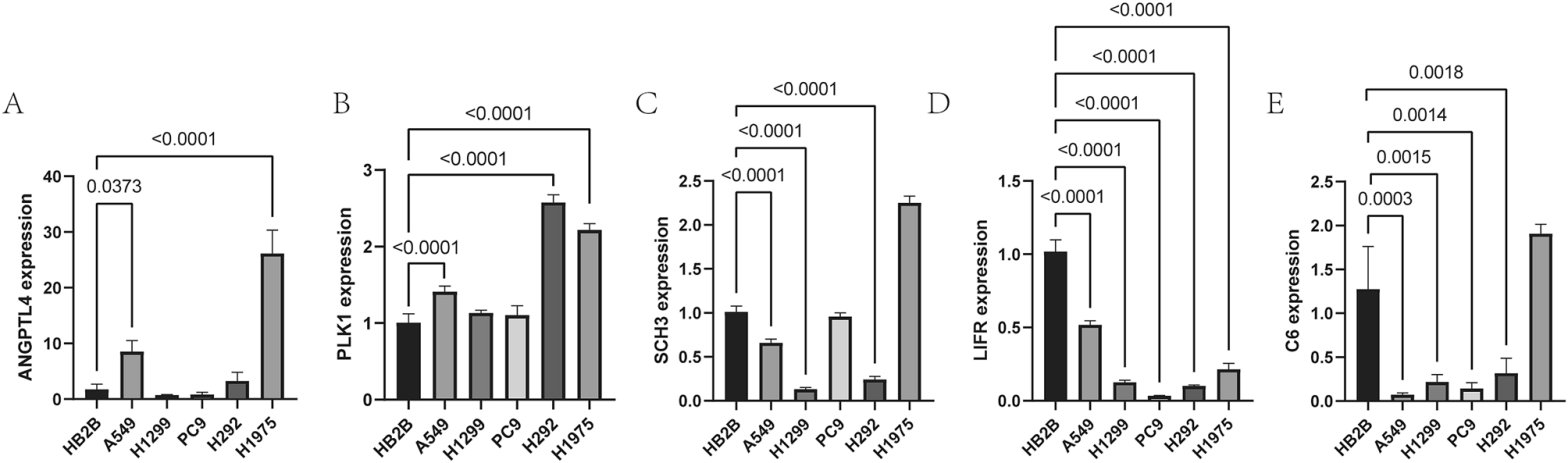
The expression levels of five genes in the construction of IGSPP. A-E. The expression levels of five genes in cell lines.

RT-qPCR was used to compare the expression levels of five genes used to construct IGSPP between five lung carcinoma cells and bronchial epithelial cell Beas-2B. Compared with the normal bronchial epithelial cell Beas-2B, the expression levels of ANGPTL4 is up-regulated in A549 and H1975 cells (Fig. 5A), PLK1 is up-regulated in A549, H292 and H1975 cells (Fig. 5B), SHC3 is down -regulated in A549 and H1299 cells (Fig. 5C), LIFR is down-regulated in all five cells (Fig. 5D), C6 is down-regulated in A549, H1299, PC9, and H292 cells (Fig. 5E). In general, the expression patterns of IGSPP genes in cultured lung carcinoma cells and LUAD tissues were consistent with TCGA data.

### IGSPP can predict the survival of LUAD patients

**Figure 6:**
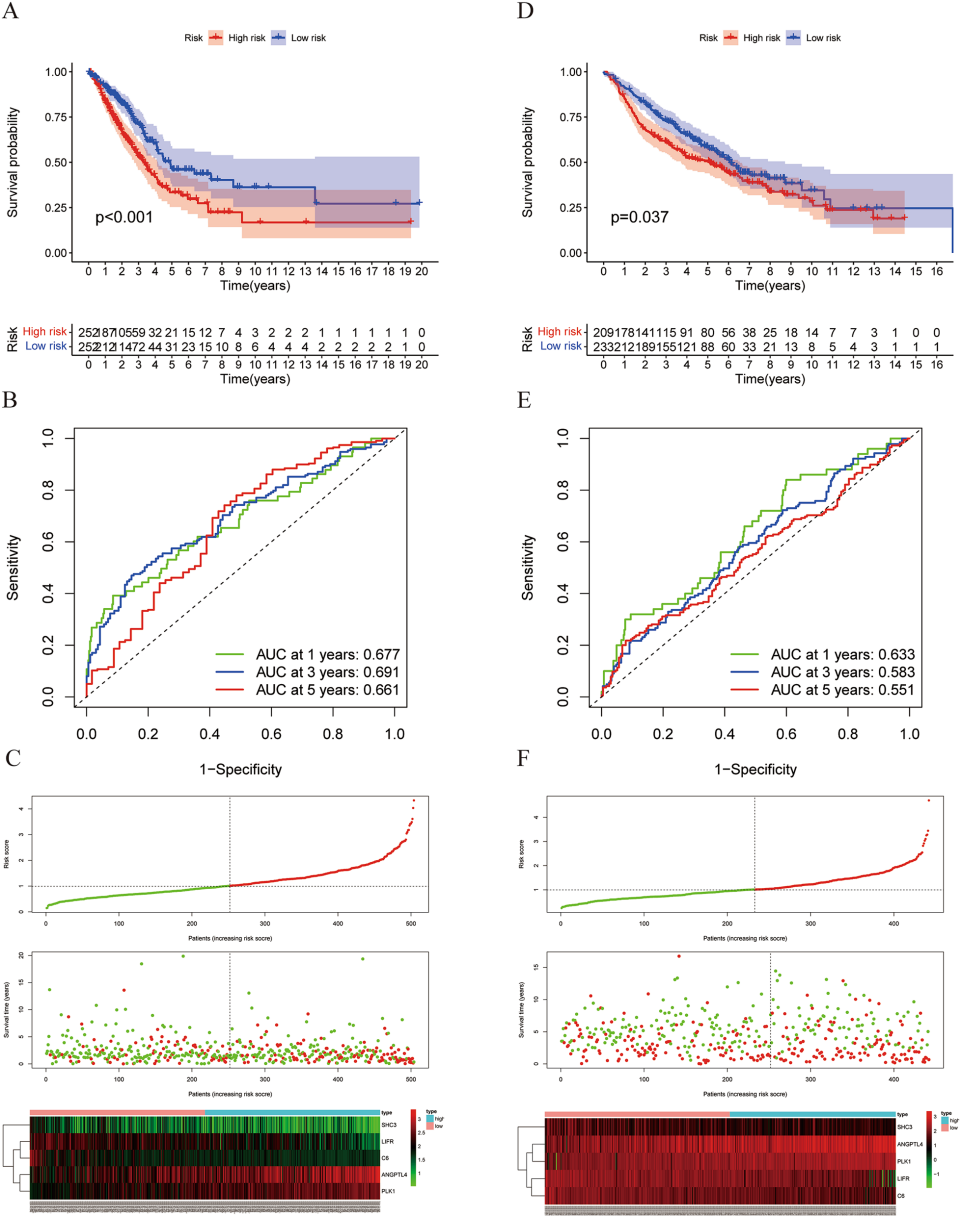
The relationship between the IGSPP scores and the prognosis of patients. (**A**, **D**) KM survival curves of IGSPP high- and low-risk groups TCGA training cohort (**A**) and GEO validation cohort (**D**). (**B**, **E**) Time ROC curves of the TCGA cohort (**B**) and GEO cohort (**E**). (**C**, **F**) (From top to bottom) patient risk score distribution, scatter diagram of patient survival status, and expression patterns of risk genes.

LUAD patients in the TCGA training cohort were divided into a low-risk group and a high-risk group according to the median risk score calculated by the IGSPP formula. As shown in Fig. 6A, the survival of the high-risk group of TCGA was significantly lower than that of the low-risk group (p < 0.001). IGSPP time-dependent ROC curves showed that the areas under the curves (AUCs) of IGSPP were 0.677, 0.691 and 0.661 for 1-, 3- and 5-year overall survival (OS), respectively (Fig. 6B). This result showed that the IGSPP we constructed had potential for monitoring survival. We ranked the risk score of each patient in the TCGA training cohort and used a dot map to show the survival status of the patient. As shown in Fig. 6C, the higher the risk score, the shorter the survival time. A heatmap describing the expression pattern of the DEIRGs in both subgroups was constructed. We further verified the predictive ability of IGSPP in the GSE68465 (Fig. 6D–F), GSE30219 (Fig. S1A–C) and GSE72094 (Fig. S1D–F) validation cohorts. The results of the validation cohort were highly consistent with those of the training cohorts. Therefore, the IGSPP had high accuracy in predicting OS in LUAD patients.

**Figure 7:**
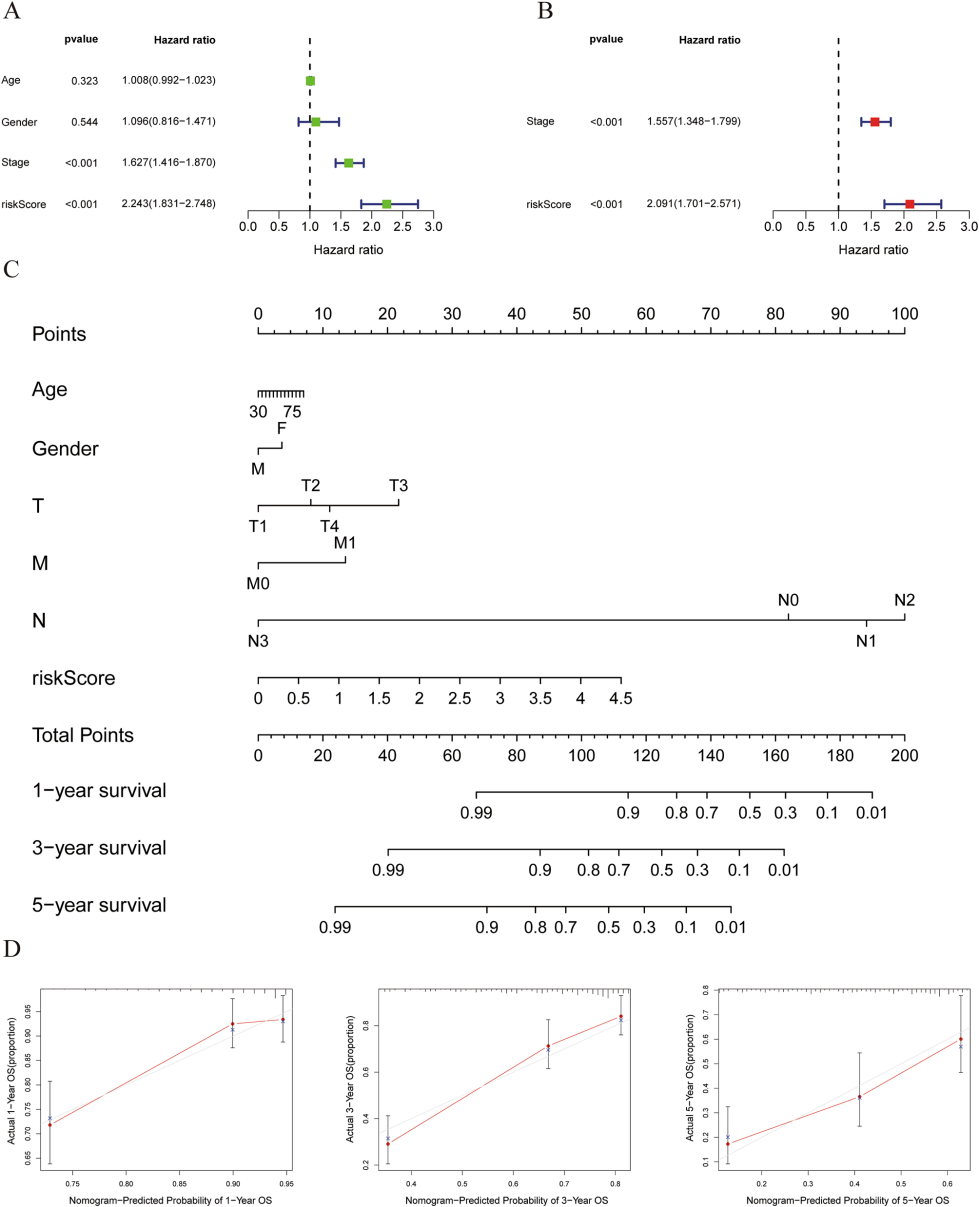
Prognostic value of IGSPP in the TCGA cohort. (**A**) Univariate Cox regression analysis. (**B**) Multivariate Cox regression analysis. (**C**) OS nomograms of 1-, 3- and 5-year. (**D**) Consistency between predicted and observed 1-, 3- and 5-year survival rates.

We evaluated the impact of clinicopathological factors and prognostic model risk scores on the OS of LUAD patients by performing univariate Cox regression analysis on the TCGA training cohort (Fig. 7A). Tumor stage and patient risk score were adverse factors of OS. We also performed multivariate Cox regression analysis (Fig. 7B). This confirmed that IGSPP could be used as an independent prognostic factor in LUAD patients after adjusting for other clinicopathological factors. To improve the prediction of survival of LUAD patients, we constructed a nomogram model of the TCGA cohort to quantitatively evaluate the OS of individual patients according to the variables related to OS (age, sex, tumor invasion, lymph node metastasis, distant metastasis and risk score) (Fig. 7C). Each patient received a total score from each prognostic parameter. The higher the total score, the lower the survival. The calibration curve of the prognostic nomogram showed good consistency between the prediction of the TCGA cohort and the actual 1-, 3- and 5-year survival rates (Fig. 7D). This result was further verified in the GSE68465 (Fig. S2A–D), GSE30219 (Fig. S3A–D) and GSE72094 (Fig. S4A–D) validation cohorts.

We used the chi-square test to analyze the correlation between IGSPP risk scores and a variety of clinicopathological factors in the TCGA cohort. Correlations between tumor pathological stage, tumor invasion, lymph node metastasis, sex and age and the risk score of the patients were observed (Fig. S5A). We also analyzed the effect of IGSPP risk scores on the prognosis of patients with different clinical characteristics. We found that the OS of patients with high risk was lower than that of patients with low risk in different stages (Fig. S5B).

### Molecular characteristics of the different groups of IGSPP

**Figure 8:**
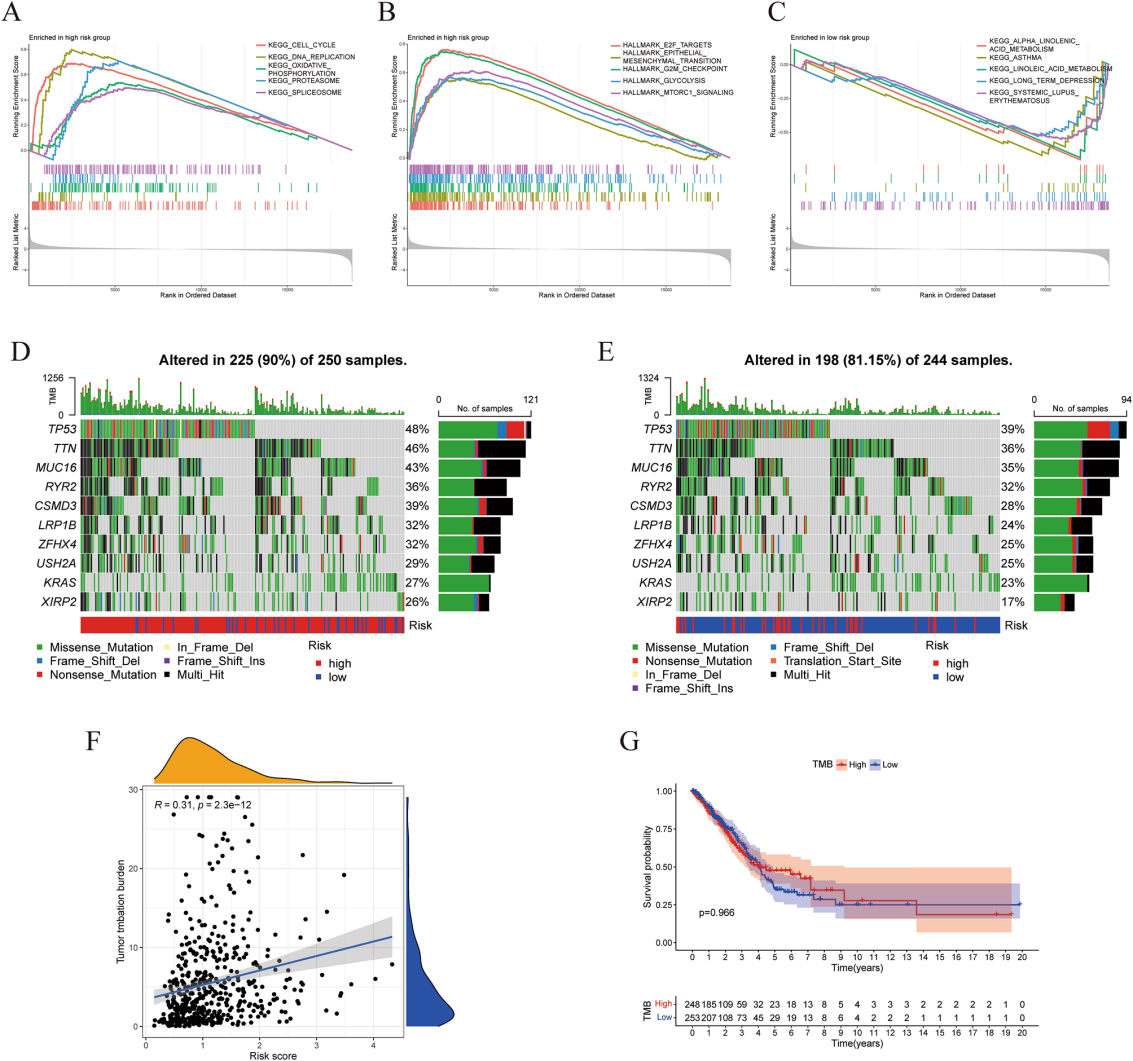
Molecular characteristics of IGSPP subgroups. (**A**, **B**) GSEA enrichment analysis in the IGSPP high-risk group (p < 0.05). (**C**) GSEA enrichment analysis in the IGSPP low-risk group (p < 0.05). (**D**) Gene mutations of patients within the IGSPP high-risk group. (**E**) Gene mutations of patients within the IGSPP low-risk group. (**F**) Correlation between the TMB and IGSPP score. (**G**) Correlation between the TMB and OS in patients with LUAD.

We carried out GSEA on different IGSPP subgroups to determine the enriched gene sets. The gene sets of the IGSPP high-risk subgroup were significantly enriched in cell cycle pathways, such as "Cell cycle", "DNA replication", "G2 M checkpoint" and "E2F targets" (Fig. 8A,B). This suggested that the cell cycle progression of LUAD patients in the high-risk IGSPP group was accelerated, which may lead to excessive proliferation of tumor cells. The gene sets in the low-risk group were significantly enriched in substance metabolism pathways (Fig. 8C). The detailed results of GSEA are listed in Tables S8 and Table S9.

Gene mutation analysis showed that the mutation rate of the IGSPP high-risk group was significantly higher than that of the IGSPP low-risk group. Missense mutations were the most frequent mutation type, followed by multipoint mutations. We visualized the top ten genes with the highest mutation rate. The mutation rates of TP53, TTN, MUC16, RYR2, CSMD3, LRP1B, ZFHX4, USH2A, KRAS, and XIRP2 were all more than 15% in both IGSPP subgroups (Fig. 8D,E). We also explored the correlation between the tumor mutation burden (TMB) and IGSPP risk score. The IGSPP risk score was positively correlated with TMB (Fig. 8F). The effect of TMB on the prognosis of LUAD patients is shown as a KM survival curve. The results showed that there was no significant correlation between TMB and OS (p = 0.966) (Fig. 8G).

### Immune characteristics of different groups of IGSPP

**Figure 9:**
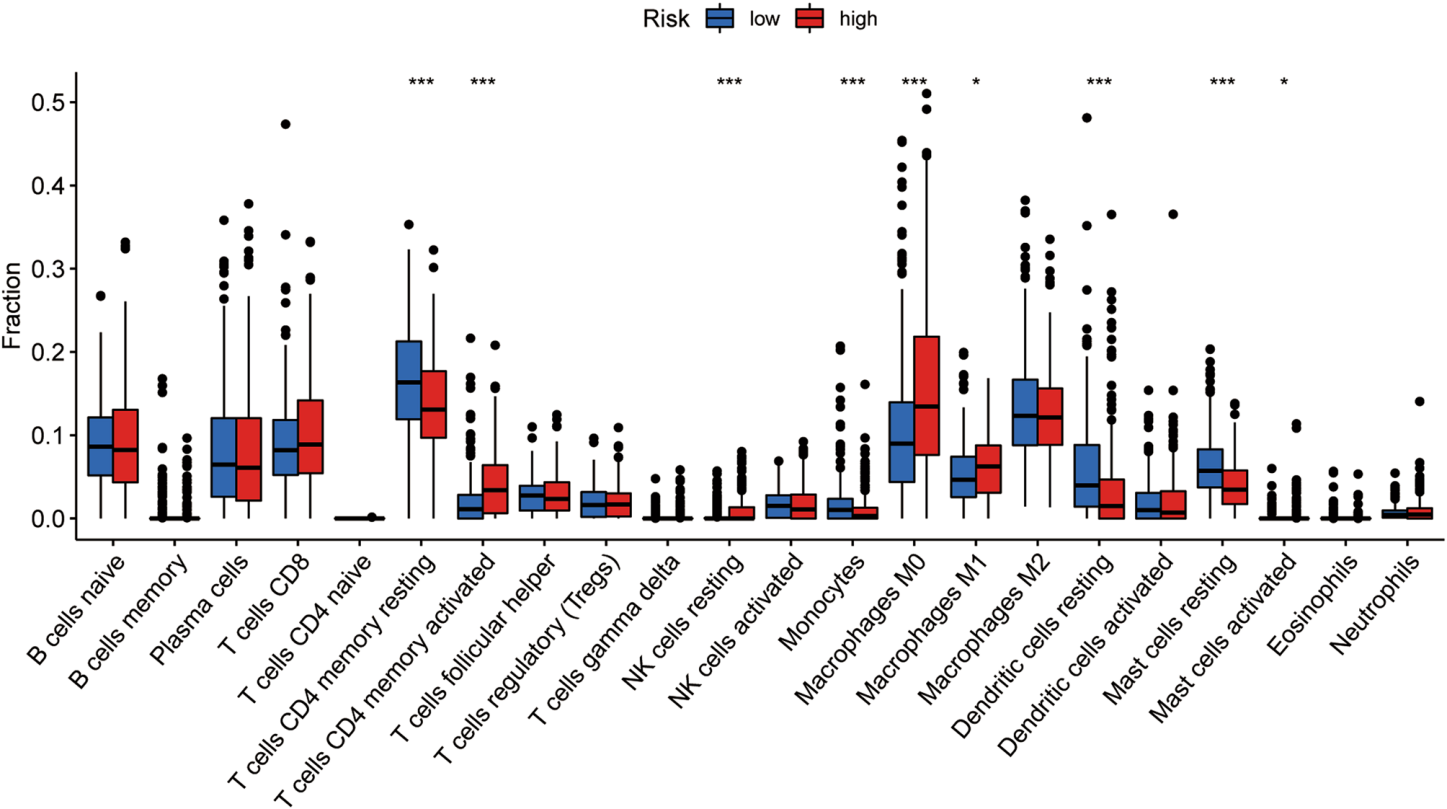
Immune cell infiltration in the IGSPP subgroups. Infiltration abundance of 22 immune cells in the IGSPP subgroups. Blue represents the low-risk group of IGSPP, and red represents the high-risk group. The horizontal line represents the median, and the bottom and top of the box are the 25th and 75th percentiles (quartile intervals), respectively. The Wilcoxon test was used to evaluate the differences between the two subgroups (*P < 0.05, **P < 0.01, ***P < 0.001, ****P < 0.0001).

CIBERSORT is a tool for assessing immune cell infiltration. We used it to assess the relative proportion of 22 immune cells in all LUAD cases. CD4 memory activated T cells, activated NK cells, M0 macrophages, M1 macrophages and activated mast cells were more abundant in the high-risk subgroup of IGSPP, while CD4 memory resting T cells, monocytes, resting dendritic cells and resting mast cells were more abundant in the low-risk subgroup of IGSPP (Fig. 9). The content of immune cells in the high-risk group of IGSPP is shown in Fig. S6A.

We also discussed the prognostic value of 22 kinds of immune cells. Among them, the infiltration abundance of naive B cells, plasma cells, resting CD4 memory T cells, activated CD4 memory T cells, regulatory T cells (T_regs_), monocytes, M0 macrophages, M1 macrophages, resting dendritic cells, activated dendritic cells, resting mast cells and neutrophils was significantly correlated with OS. The higher the infiltration abundance of naive B cells, resting dendritic cells, resting mast cells, monocytes, plasma cells, resting memory CD4 T cells and regulatory T cells (T_regs_), the better the prognosis, while the higher the infiltration abundance of activated dendritic cells, M0 macrophages, M1 macrophages, neutrophils and memory CD4 T cells, the worse the prognosis (Fig. S6B). It seems that IGSPP is related to the infiltration levels of most immune cells and that IGSPP reflects the state of the tumor immune microenvironment.

**Figure 10:**
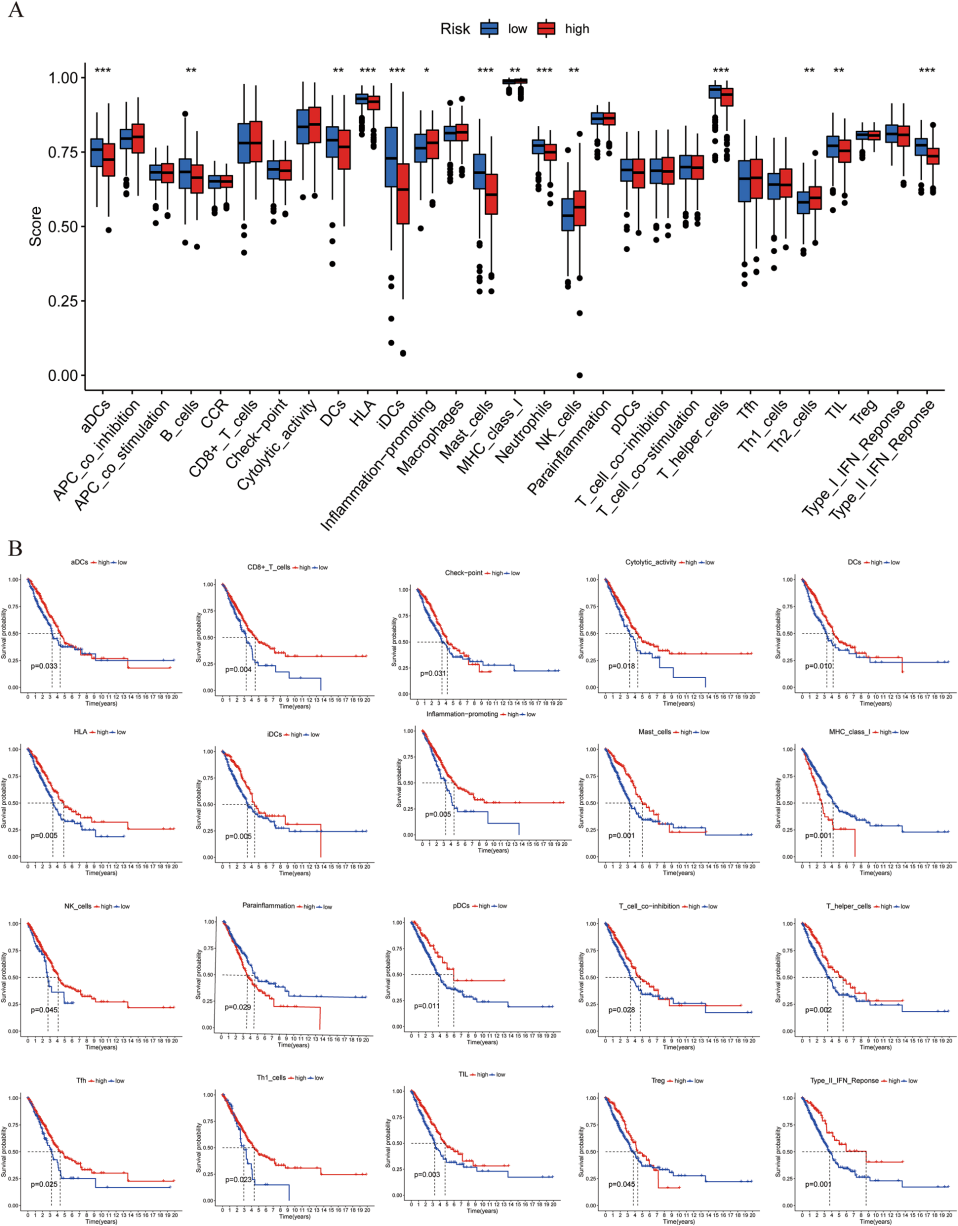
Immune characteristics of the IGSPP subgroups. (**A**) Immune-related functions were enriched and analyzed by ssGSEA and then compared among different IGSPP subgroups. The scattered points represent the ssGSEA scores of the two subgroups, the horizontal line represents the median, and the bottom and top of the box represent the 25th and 75th percentiles (quartile intervals), respectively. The Wilcoxon test was used to evaluate the difference between two subgroups (*p < 0.05, **P < 0.01, ***P < 0.001). (**B**) Correlation between immune-related function score and overall survival time.

We used ssGESA to analyze the differences in immune cells and functions among the IGSPP subgroups. As shown in Fig. 10A, inflammation-promoting, MHC class I, NK cells and Th2 cells had higher scores in the IGSPP high-risk group, while aDCs, B cells, DCs, HLA, iDCs, mast cells, neutrophils, T helper cells, TILs and type II IFN response had higher scores in the IGSPP low-risk group. We also explored the effects of immune functions on prognosis. In a total of 20 immune cells and functions related to prognosis, 12 immune cells include aDCs, B cells, CD8+ T cells, DCs, iDCs, Mast_cells, NK_cells, pDCs, T_helper_cells, Tfh, Th1_cells, TIL and Treg, and 6 immune functions include Check-point, Cytolytic_activity, HLA, Inflammation-promoting, T cell co-inhibition and Type II IFN Response had a positive correlation with the prognosis of LUAD patients, while the other 2 immune functions of MHC_class_I and Parainflammation had a negative correlation with the prognosis (Fig. 10B). This result is consistent with the previous analysiswhich showed IGSPP was associated with the level of most immune cell infiltration and immune function, and means that our IGSPP can be used to reflect the state of the tumor microenvironment.

Six immune subtypes of cancer, including C1 (wound healing), C2 (IFN-γ dominant), C3 (inflammatory), C4 (lymphocyte depleted), C5 (immunologically quiet) and C6 (TGF-γ dominant), were identified to define immune response patterns impacting the prognosis^28^. We analyzed the distribution of various immune subtypes in different IGSPP subgroups by the chi-square test. The results showed that C1 and C2 subtypes accounted for more in the high-risk subgroup of IGSPP. The content of Th2 cells in the C1 subtype was higher^28^, and Th2 cells could promote the growth of tumor cells^29^, indicating that the high-risk subgroup of IGSPP had a higher tumor proliferation rate. The proportion of C3 subtype in the low-risk subgroup of IGSPP is significantly higher than that in the high-risk subgroup. The C3 subtype is defined by the elevated Th17 and Th1 genes^28^. Th1 has an inhibitory effect on tumor growth^29^. It indicates that the low-risk subgroup of IGSPP should have a lower tumor growth rate (Fig. S7). The above results further verify that the patients in the high-risk subgroup of IGSPP analyzed in our previous analysis have a worse prognosis.

### Benefits of ICIs treatment in IGSPP subgroups

**Figure 11:**
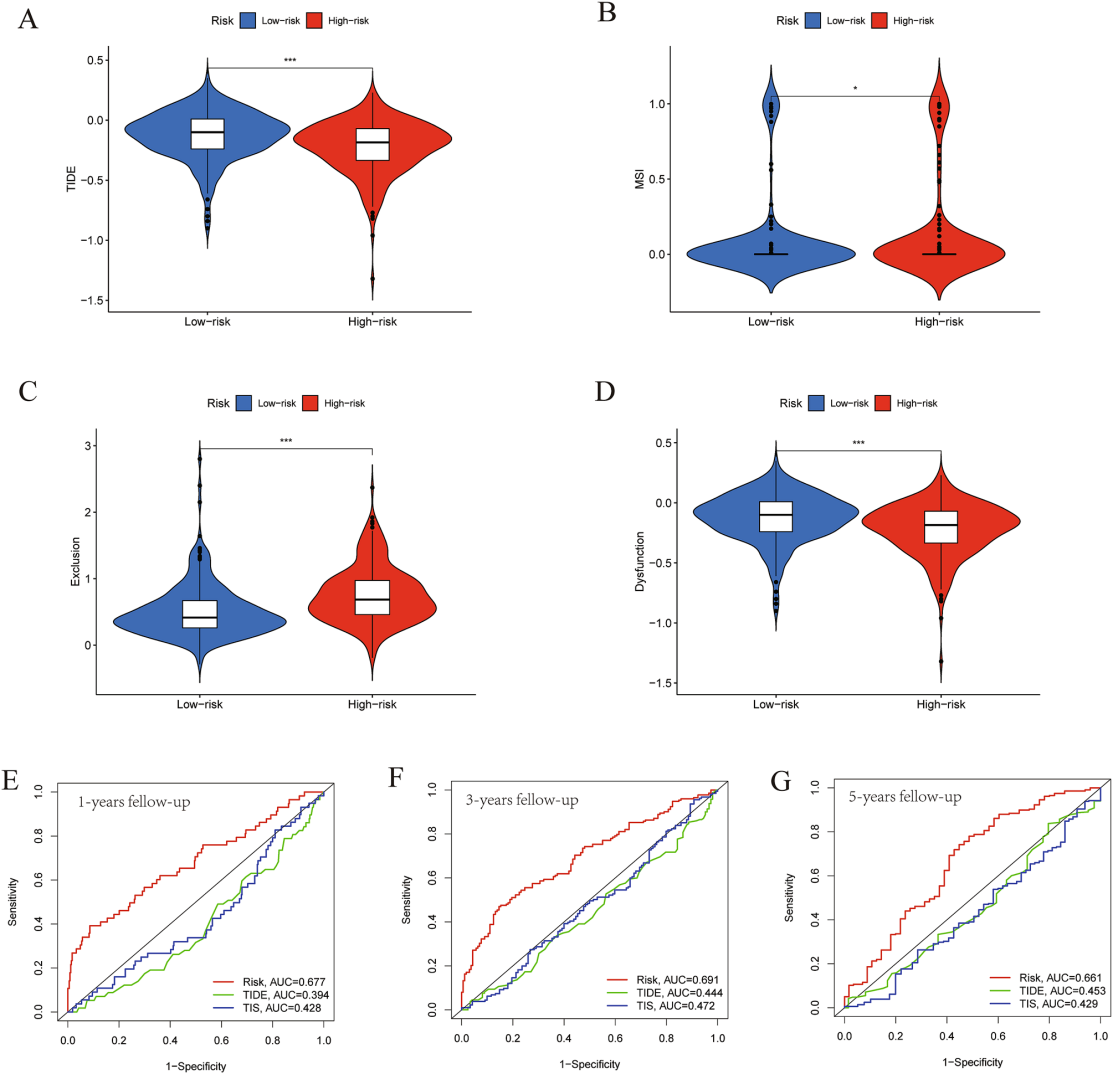
The prognostic value of IGSPP for ICI treatment. (**A**–**D**) Scores of TIDE, MSI, T cell exclusion and T cell dysfunction in different IGSPP groups. The Wilcoxon test was used to evaluate the difference (*P < 0.05, **P < 0.01, ***P < 0.001). (**E**–**G**) The 1-, 3- and 5-year ROC curves based on the IGSPP, TIS and TIDE scores of the TCGA cohort.

We used tumor immune dysfunction and exclusion (TIDE) to evaluate the potential clinical efficacy of ICIs therapy in IGSPP subgroups^30^. The results showed that the TIDE score of the IGSPP high-risk subgroup was lower than that of the low-risk subgroup. This means that patients within the IGSPP high-risk group would benefit more from ICIs treatment than those in the IGSPP low-risk group (Fig. 11A). In addition, we analyzed the relationship between the IGSPP subgroups and microsatellite instability (MSI), T cell exclusion and T cell dysfunction scores. We found that microsatellite instability (MSI) and T cell exclusion scores were higher in the high-risk IGSPP group (Fig. 11B,C), while T cell dysfunction scores were higher in the low-risk IGSPP group (Fig. 11D).

The tumor inflammation signature (TIS) measures pre-existing but suppressed adaptive immune responses in tumors with an 18-gene signature, including genes associated with antigen presentation, chemokine expression, cytotoxic activity, and adaptive immune resistance^31^. We extracted TIS gene expression from TCGA LUAD patients and drew 1-, 3- and 5-year ROC curves based on TIS and TIDE scores. We compared these curves with our IGSPP time ROC curves. As shown in Fig. 11E–G, the predictive ability of IGSPP was significantly better than those of TIDE and TIS in the TCGA cohort.

### Expressions of different immune checkpoints in IGSPP subgroups

**Figure 12:**
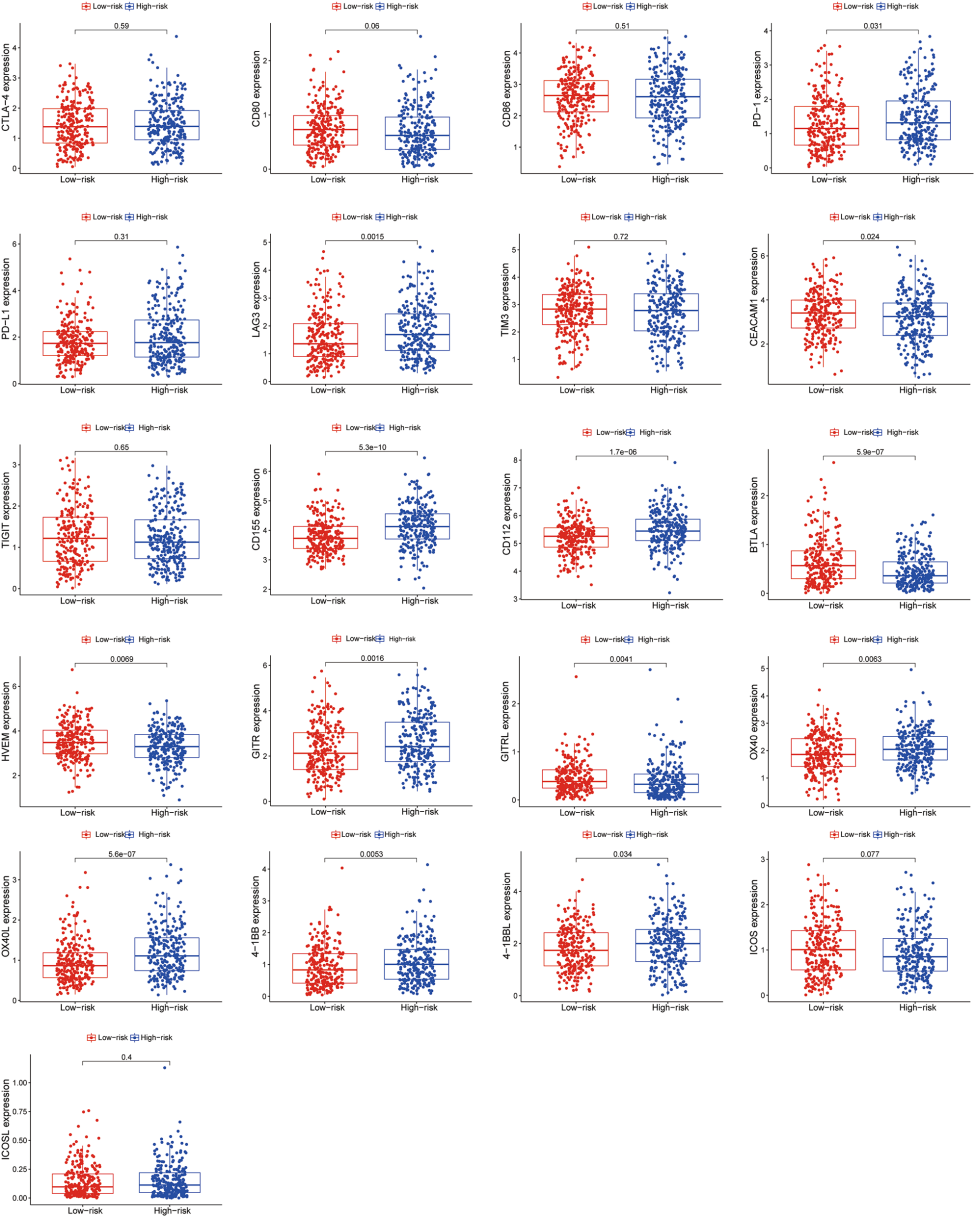
The expression of some key immune checkpoint molecules.

We compared the expressions of a group of co-stimulatory and/or co-inhibitory receptors of T cells as well as their ligands between IGSPP subgroups by using the TCGA database LUAD transcriptome expression data. As shown in Fig. 12, the mRNA expressions of co-inhibitory receptors/ligands, such as PD-1, LAG3, CD155, CD112, and BTLA, and co-stimulatory receptors/ligands, such as GITR, OX40, OX40L, 4-1BB, and 4-1BBL were higher in the high-risk subgroup. These results further suggested that patients in the high-risk subgroup should be more likely to benefit from ICIs therapy, not only in PD-1/PD-L1 treatment, but also in agents targeting emerging immune checkpoints, such as LAG3, GITR and OX40.

## Discussion

The oncogenesis and development of lung cancer depend not only on the changes in cancer genomes and the molecular characteristics of cancer cells but also on their interaction with the tumor environment, especially with the immune system^6^. Clinical applications of immune checkpoint inhibitors (ICIs), especially PD-1/PD-L1 antibodies, have improved the treatment of a variety of cancers, including NSCLCs^32,33^. The development of ICIs is also considered to be a milestone in immune oncology. However, the proportion of LUAD patients benefiting from ICIs is 25% lower than that of LUSC patients^34^. Novel biomarkers have been studied to predict the response of LUAD patients to ICIs therapy and further promote accurate immunotherapy^35^.

In this study, we integrated TCGA expression data and immune-related gene data and obtained 681 DEIRGs (Fig. 1B). Then, we used WGCNA and Cox regression analysis to screen DEIRGs related to the prognosis of LUAD patients. Finally, five genes were identified by LASSO and multivariate Cox regression analysis (Fig. 4B–D). These genes were used to construct the IGSPP. The prognostic value of the IGSPP was assessed in the TCGA training cohort and GEO validation cohort. The prognosis of patients within the IGSPP low-risk subgroup was better than that of patients within the IGSPP high-risk subgroup (Fig. 6A,D). IGSPP has been shown to be an immune-related biomarker. Its predictive value is reliable and accurate.

Five genes, including ANGPTL4, LIFR, SHC3, PLK1 and C6, were used to construct the IGSPP. Among them, ANGPTL4 and PLK1 are adverse prognostic genes in LUAD patients, while LIFR, SHC3 and C6 are favorable prognostic genes. ANGPTL4 has been identified as an oncogene in papillary thyroid carcinoma (PTC). Upregulation of ANGPTL4 promotes PTC cell proliferation and reduces PTC cell cycle arrest^36^. Overexpression of PLK1 promotes the proliferation of renal cell carcinoma (RCC) cells and inhibits apoptosis^37^. It also promotes the migration and invasion of NSCLC cells, thus reducing the survival rate of NSCLC patients^38^. PLK1 has also been confirmed as a key regulatory protein of the cell cycle^39^. In contrast, overexpression of LIFR in hepatocellular carcinoma (HCC) and breast cancer (BC) inhibited cancer cell migration and invasion in vivo and in vitro^40,41^. SHC3 has been used to construct other prognostic biomarkers in NSCLC^42^. NSCLC patients with higher expression of SHC3 have a better prognosis. This result is in agreement with our analysis. However, a higher expression level of SHC3 in HCC promotes the migration and invasion of HCC cells, leading to a worse prognosis of HCC patients^43^. It seems that SHC3 has different functions in various tumors. C6 is the sixth component of complement. Patients with C6 deficiency are susceptible to infection with *Neisseria meningitidis*^44^. C6 deficiency is common among African Americans in the southeastern United States^45^. However, there are few studies on the functions of complement C6 in lung cancer.

We verified the expression levels of five genes used to construct IGSPP in cultured lung cancer cell lines (Fig. 5A–E). The expression levels of these five genes in lung cancer cell lines are consistent with the results of our analysis. Since the expression levels of genes in each lung cancer cell line are different, the expression levels of genes in these five lung cancer cell lines are not all the same.

Abnormal cell proliferation is one of the most important phenomena of cancer^46^. Recent studies have shown that cell cycle-related genes are strongly associated with the prognosis of many malignant tumors. It is possible to evaluate tumor immunogenicity and response to ICIs therapy by analyzing cell cycle-related genes^47^. In this study, two genes (ANGPTL4 and PLK1) of the IGSPP that we constructed were involved in the regulation of the tumor cell cycle^36,39^. GSEA of the IGSPP subgroups showed that genes in the IGSPP high-risk group were significantly enriched in cell cycle-related pathways (Fig. 8A–B). These results suggest that the predictability and accuracy of the IGSPP in predicting the OS of LUAD patients are reasonable in the context of the molecular mechanism of cancer.

To further understand the immune characteristics of the different IGSPP subgroups, we analyzed gene mutations in the IGSPP subgroups (Fig. 8D–E). We found that missense mutations were the most common in LUAD patients’ genomes, followed by multiple point mutations. Several genomic alterations, such as alterations in EGFR, KRAS, STK11, ALK, JAK2, TP53, and ATM, have been shown to be associated with ICI efficacy. Among these mutations, co-occurring mutations in TP53 and other genes (EGFR, STK11, or KRAS) have been shown to have predictive value for immune checkpoint inhibitors^10,48^. Among the top 10 genes with the highest mutation rates we visualized, TP53 is the gene with the highest mutation frequency, and its mutation frequency is higher in the IGSPP high-risk subgroup than in the low-risk subgroup. It has been reported that mutant TP53 is an adverse prognostic factor of advanced NSCLC and head and neck squamous cell carcinoma (HNSCC)^23,49^. This result is consistent with our observation of a poor prognosis (OS) in the IGSPP high-risk subgroup. However, some studies have reported that mutant TP53 promotes antitumor immunity. For example, the antitumor immune signatures of TP53-mutated cancer are significantly higher than those of TP53 wild-type cancer in LUAD and breast invasive carcinoma (BRCA)^50^. This suggests that patients in the IGSPP high-risk subgroup should have better responses to immunotherapy. Other studies have shown that LUSC patients with high TP53 mutation rates had shorter overall survival times but were more responsive to ICIs treatment^51^. We also observed that the mutation rate of KRAS in the high-risk subgroup of IGSPP was also higher than that in the low-risk subgroup, with a mutation rate higher than 20%. KRAS has a high mutation rate in LUAD (20%-40%) and is associated with poor patient prognosis^52,53^. However, recent studies have shown that NSCLC patients with KRAS mutations have different degrees of sensitivity to ICIs treatment, and KRAS mutations are significant in enhancing PD-L1 expression, promoting T cell infiltration, and enhancing tumor immunogenicity^52,54,55^. Other common gene mutations associated with ICIs treatment, such as EGFR, STK11, ALK, JAK2, and ATM, had low mutation rates in this study. Considering that ICIs therapy had been advocated as standard second-line therapy for NSCLC patients without EGFR/ALK genetic alteration^56,57^, the cohorts in our study should not have contained many patients with EGFR/ALK mutations. The co-mutation of TP53 and KRAS suggests that the high-risk subgroup of IGSPP is more responsive to ICIs treatment. LUAD with KRAS and TP53 mutations shows a higher TMB^58,59^. We explored the correlation between IGSPP and known immunotherapy biomarkers. The IGSPP scores were positively correlated with TMB but not significantly correlated with the expression of PD-L1 and CTLA-4. NSCLCs usually have higher mutation burdens and can stimulate a stronger antitumor immune response (Fig. 8F). TMB can preferentially predict the clinical benefits of NSCLC immunotherapy regardless of the expression of PD-L1^60,61^. This also suggests that the high-risk group of IGSPP may benefit more from ICIs therapy.

The role of immune cell infiltration in tumors has increasingly attracted attention in recent years. It has been shown that immune cell infiltration plays key roles in tumor progression and the response to immunotherapy in LUAD and colorectal cancer (CRC)^62,63^. In this study, we explored the potential relationship between IGSPP and immune cell infiltration (Fig. 9). We found that CD4+ T cells, NK cells, M0 macrophages, M1 macrophages and mast cells were enriched in the IGSPP high-risk subgroup^23^. This result is consistent with other immune gene prognostic index studies in HNSCC, which also showed that CD4+ T cells and M1 macrophages were abundant in the high-risk group. Cancer immunotherapy promotes the activity of cytotoxic T lymphocytes (CTLs) in tumors and assists in the activation of tumor-specific CTLs in lymphoid organs, establishing efficient and lasting antitumor immunity. CD4+ T cells enhance the antitumor activity of CTLs^64^. M1 proinflammatory macrophages can engulf tumor cells^65^. Macrophage-directed cancer immunotherapy, which modulates M2/repair-type macrophages into M1/kill-type macrophages, has been shown to slow or stop cancer growth^66,67^. These results suggest that patients within the IGSPP high-risk subgroup might have better responses to immunotherapy.

The tumor immune dysfunction and exclusion (TIDE) algorithm simulates two main immune escape mechanisms of tumors to predict the ICIs response^23,30^. The TIDE prediction score is positively correlated with the possibility of tumor immune escape. This indicates that patients with higher TIDE scores are less likely to benefit from ICIs therapy. In our study, the high-risk IGSPP subgroup had lower TIDE scores and higher microsatellite instability (MSI) and T cell exclusion scores, while the low-risk IGSPP subgroup had higher TIDE and T cell dysfunction scores (Fig. 11A–D). Therefore, patients with a low risk of IGSPP may have immune escape due to T cell dysfunction and have a poor response to ICIs treatment, while patients with a high risk of IGSPP could benefit more from ICIs treatment. Anti-PD-1/PD-L1 therapy for tumors with high MSI has shown lasting responses in prostate cancer and CRC^68,69^. Therefore, patients in the IGSPP high-risk group might have a better response to ICIs therapy because of MSI. Both TIDE and TIS could be used to predict the response of patients to immunotherapy^30,31^, but their foci were T cell functions and status and did not fully reflect the complexity of the TME involved in immunotherapy^23^. In addition, both markers focused on the patient's response to immunotherapy rather than the patient's survival time^23^. The advantages of our study are that IGSPP can distinguish the predicted survival time, molecular characteristics and the immune characteristics of patients.

The blockade of immune checkpoints can reduce the immune escape of tumor cells and activate immune reaction in tumor microenvironment^70^. At present, clinical trials of immune checkpoint blockade treatments mainly focus on the interaction between CTLA-4 and its ligands CD80/CD86, and the binding of PD-1 and PD-L1^71,72^. However, compared with LUSC patients, LUAD patients have lower benefits from CTLA4 and anti-PD-1 or anti-PD-L1 therapy. Expanding ICI-based treatment approaches beyond CTLA4 and PD-1/PD- L1 pathways is therefore a clear clinical need. Antibodies targeting the co-inhibitory receptors of T cells, such as LAG3, TIM3, TIGIT and BTLA, as well as agonists of the co- stimulatory receptors, such as GITR, OX40, 4-1BB and ICOS, are undergoing clinical trials^73–75^. In this study, we analyzed the mRNA expression levels of these co-inhibitory receptors and their ligands by using the TCGA database LUAD transcriptome expression data (Fig. 12). It showed that the expressions of PD-1, LAG3, CD155, CD112, and BTLA, were increased in the high-risk subgroup of IGSPP. Co-stimulatory T cell receptors play critical roles in T cell activation, differentiation, effector function and survival^75^. Activating co-stimulatory T cell receptors is promising therapeutic strategy in clinical practice^72,75^. We analyzed the mRNA expression levels of targeted costimulatory receptors GITR, OX40, 4-1BB, ICOS and their ligands (Fig. 12). It showed that the expressions of GITR, OX40, OX40L, 4-1BB, 4-1BBL were higher in the high-risk IGSPP subgroup. It suggests that our IGSPP high-risk group should be also more suitable for co- stimulatory receptors targeting therapy.

In summary, we constructed an IGSPP that can be used to predict the survival rate, molecular and immune characteristics of LUAD patients and the benefits of ICIs treatment. The predictive ability of this prognostic index in LUAD needs to be further validated to determine whether it can improve patient outcomes and treatment responses by tailoring treatment to each individual patient.

## Supplementary Information

Supplementary Legends.

Supplementary Figures.

Supplementary Tables.

## Data Availability

The datasets analyzed for this study can be found in the TCGA-LUAD project (https://portal.gdc.cancer.gov) and GEO (https://www.ncbi.nlm.nih.gov/geo/query/acc.cgi?acc=GSE68465/GSE30219/GSE72094).
