# Supplementary material for: An immune gene signature to predict prognosis and immunotherapeutic response in lung adenocarcinoma
Source: Sci Rep. 2022 May 17;12:8230. doi: 10.1038/s41598-022-12301-6 (PMC9114138; doi:10.1038/s41598-022-12301-6)
Supplement: Supplementary file 1 — Supplementary Legends. [file 41598_2022_12301_MOESM1_ESM.docx]

**Figure S1**.**Validation of IGSPP scores in GSE30219 and GSE72094 set.**

A and D. KM survival curves of IGSPP high- and low-risk groups GSE30219 validation cohort (A) and GSE72094 validation cohort (D). B and E. Time ROC curves of the GSE30219 validation cohort (B) and GSE72094 validation cohort (E). C and F. (from top to bottom) Patient risk score distribution, scatter diagram of patient survival status, and expression patterns of risk genes.

**Figure S2. Prognostic value of IGSPP in the GSE68465 validation cohort**

A. Univariate Cox regression analysis. B. Multivariate Cox regression analysis. C. OS nomograms of 1-, 3- and 5-year. D. Consistency between predicted and observed 1-, 3- and 5-year survival rates.

**Figure S3. Prognostic value of IGSPP in the GSE30219 validation cohort**

A. Univariate Cox regression analysis. B. Multivariate Cox regression analysis. C. OS nomograms of 1-, 3- and 5-year. D. Consistency between predicted and observed 1-, 3- and 5-year survival rates.

**Figure S4. Prognostic value of IGSPP in the GSE72094 validation cohort**

A. Univariate Cox regression analysis. B. Multivariate Cox regression analysis. C. OS nomograms of 1-, 3- and 5-year. D. Consistency between predicted and observed 1-, 3- and 5-year survival rates.

**Figure S5. Relationship of the IGSPP subgroups and multiple clinicopathological factors**

A. Analysis of clinical information in the TCGA cohort and different IGSPP groups. B. Correlation between OS of the TCGA cohort and clinicopathological factors.

**Figure S6. Immune cell infiltration and survival analysis**

**A.** Contents of immune cells in the high-risk and low-risk IGSPP groups. B. The relationship between immune cell infiltration and overall survival of TCGA LUAD patients.

**Figure S7. Distribution of immune subtypes in the IGSPP subgroups.**

**Table S1.** **Primer for RT-Qpcr**

**Table S2. 8191 differentially expressed genes (DEGs)**

**Table S3. 681 differentially expressed immune-related genes (DEIRGs)**

**Tables S4. Results of GO enrichment analysis of DEIRGs**

**Tables S5. Results of KEGG pathway enrichment analysis of DEIRGs**

**Tables S6. Results of GO enrichment analysis for 263 genes**

**Tables S7. Results of KEGG pathway enrichment analysis for 263 genes**

**Tables S8. GSEA analysis of KEGG subsets of canonical pathways in IGSPP subgroups**

**Tables S9.** **GSEA analysis of hallmark gene sets in IGSPP subgroups**
