# Supplementary figures and images for: An immune gene signature to predict prognosis and immunotherapeutic response in lung adenocarcinoma
Source: Sci Rep. 2022 May 17;12:8230. doi: 10.1038/s41598-022-12301-6 (PMC9114138; doi:10.1038/s41598-022-12301-6)

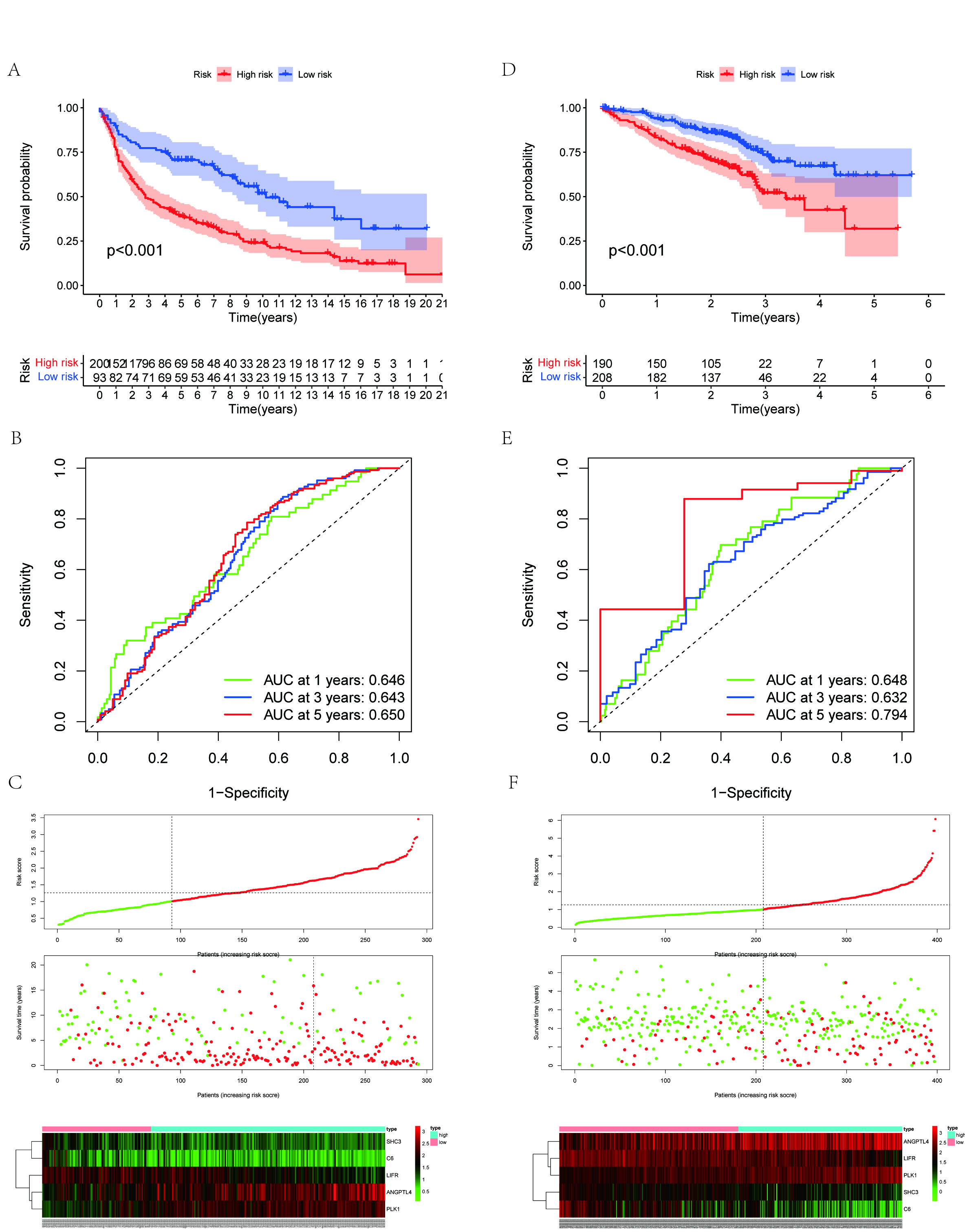

Supplement: Supplementary file 2 — Supplementary Figures. [file 41598_2022_12301_MOESM2_ESM.zip › Supplementary Figures/Figure S1.tif]

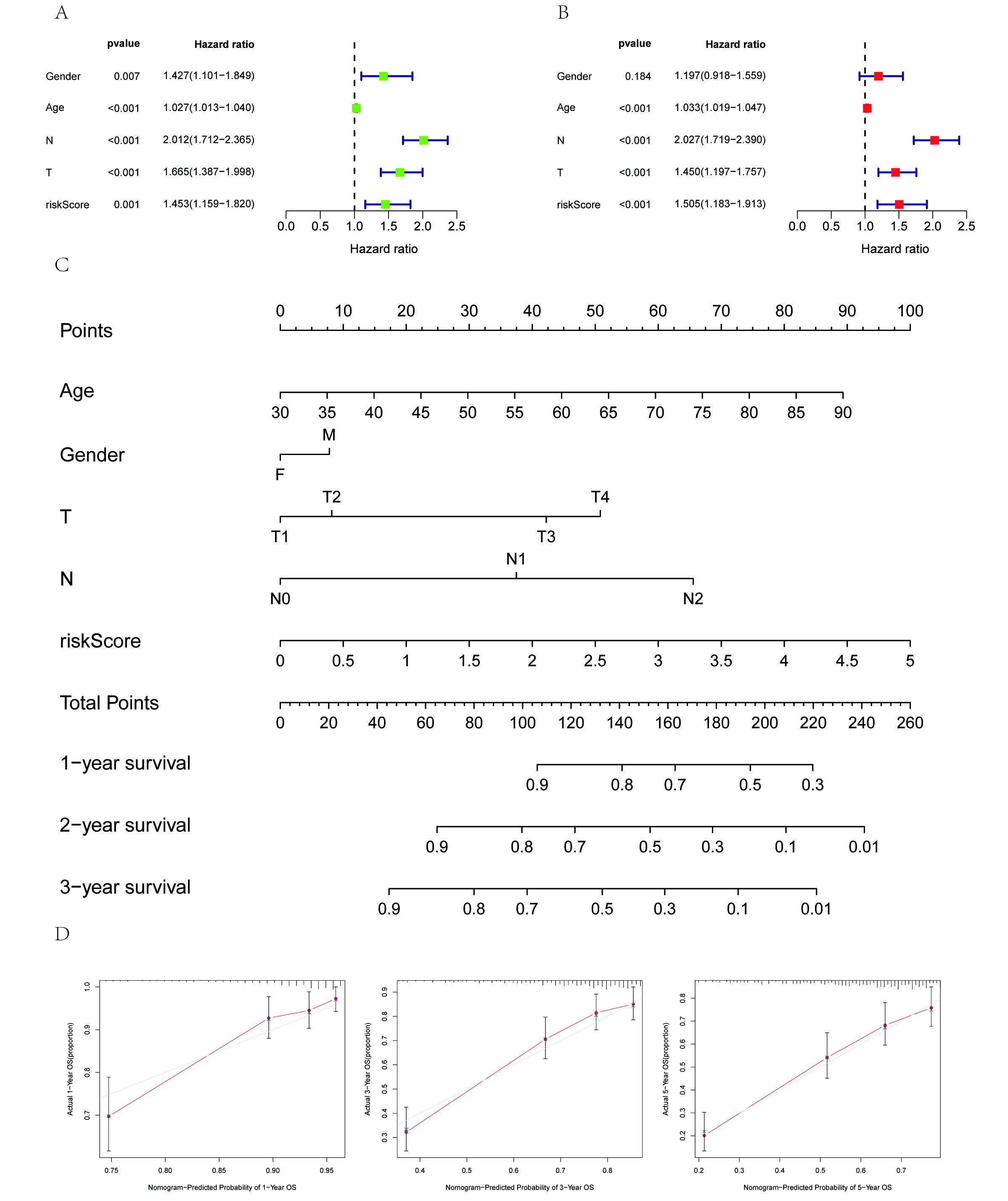

Supplement: Supplementary file 2 — Supplementary Figures. [file 41598_2022_12301_MOESM2_ESM.zip › Supplementary Figures/Figure S2.tif]

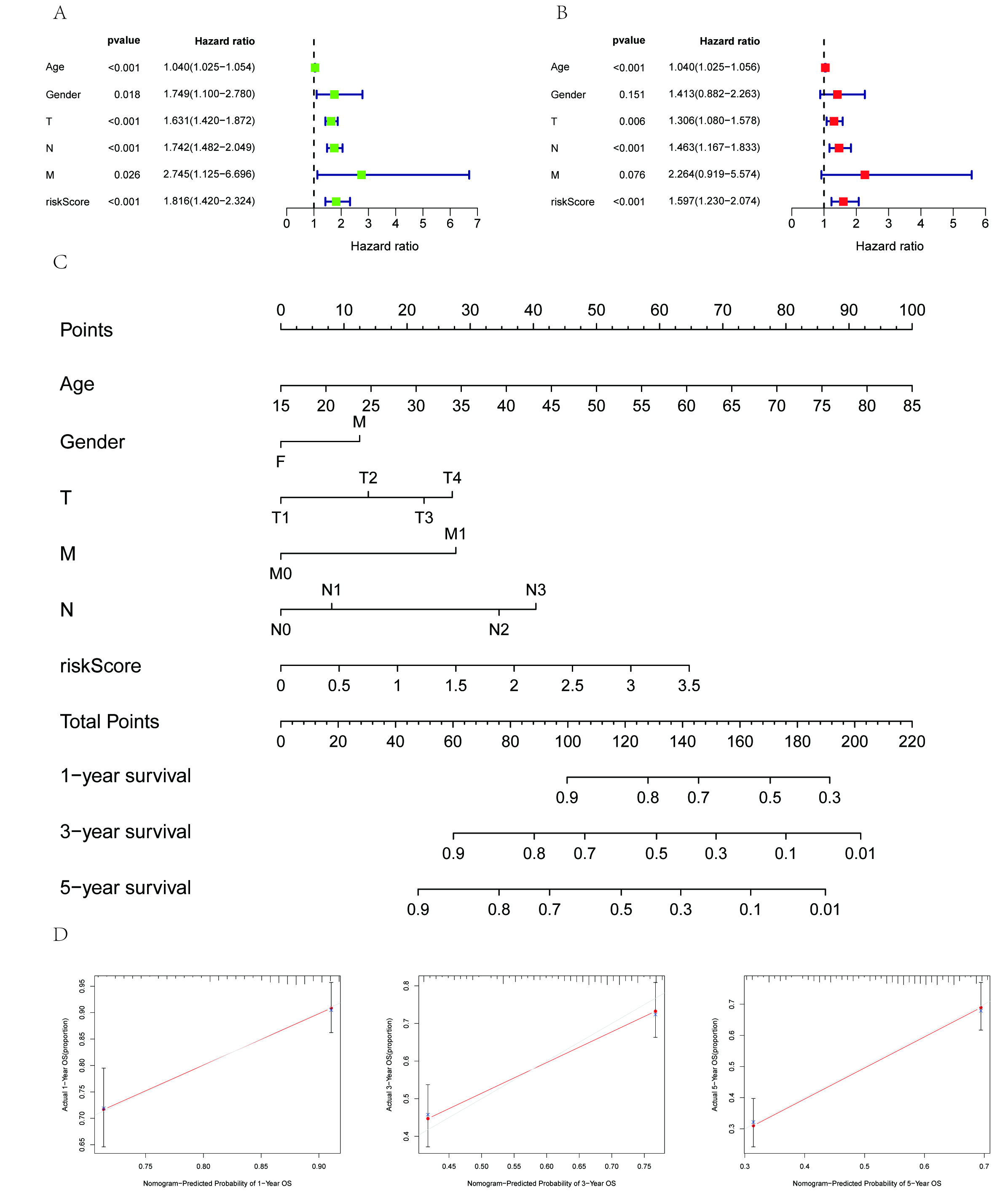

Supplement: Supplementary file 2 — Supplementary Figures. [file 41598_2022_12301_MOESM2_ESM.zip › Supplementary Figures/Figure S3.tif]

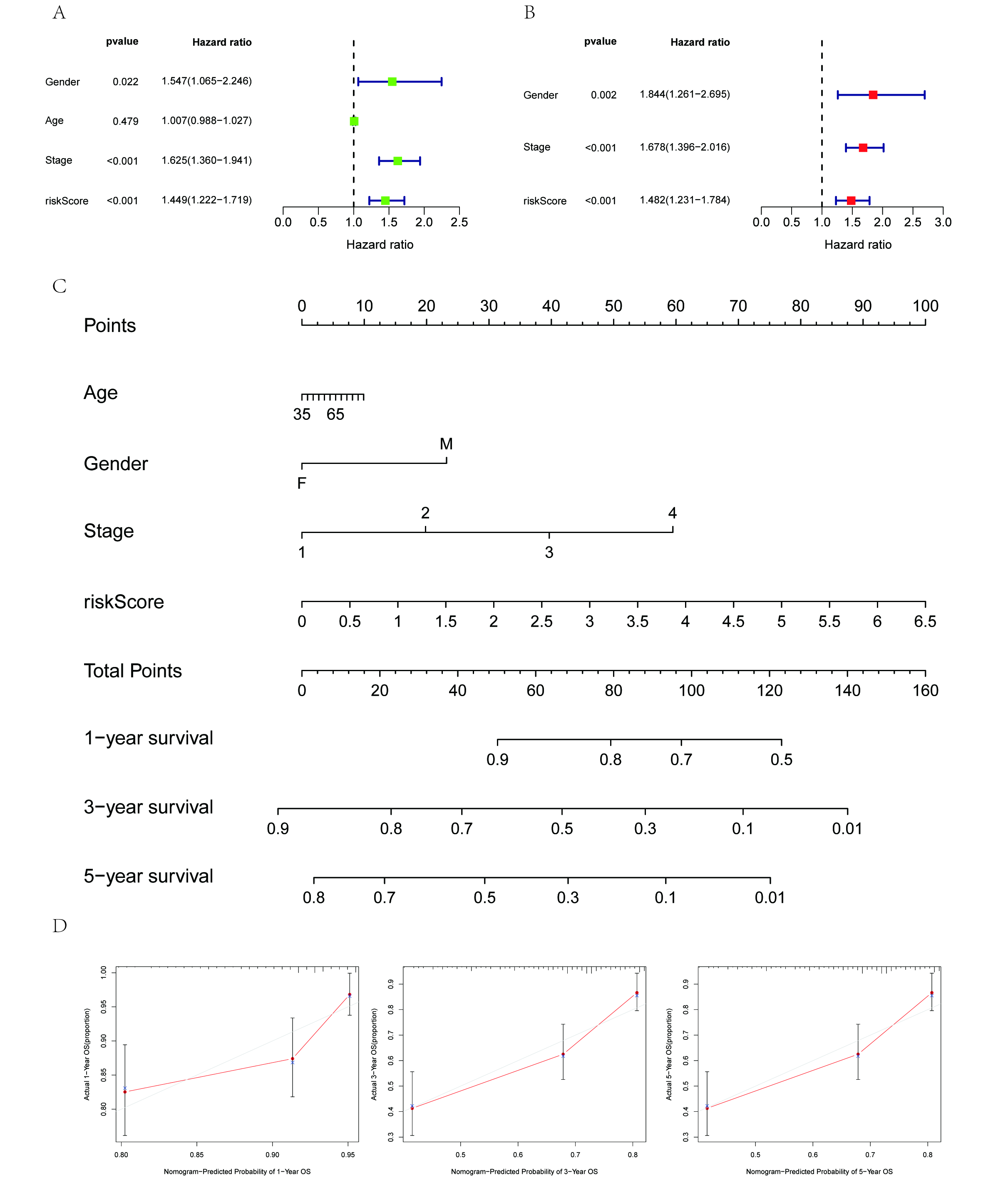

Supplement: Supplementary file 2 — Supplementary Figures. [file 41598_2022_12301_MOESM2_ESM.zip › Supplementary Figures/Figure S4.tif]

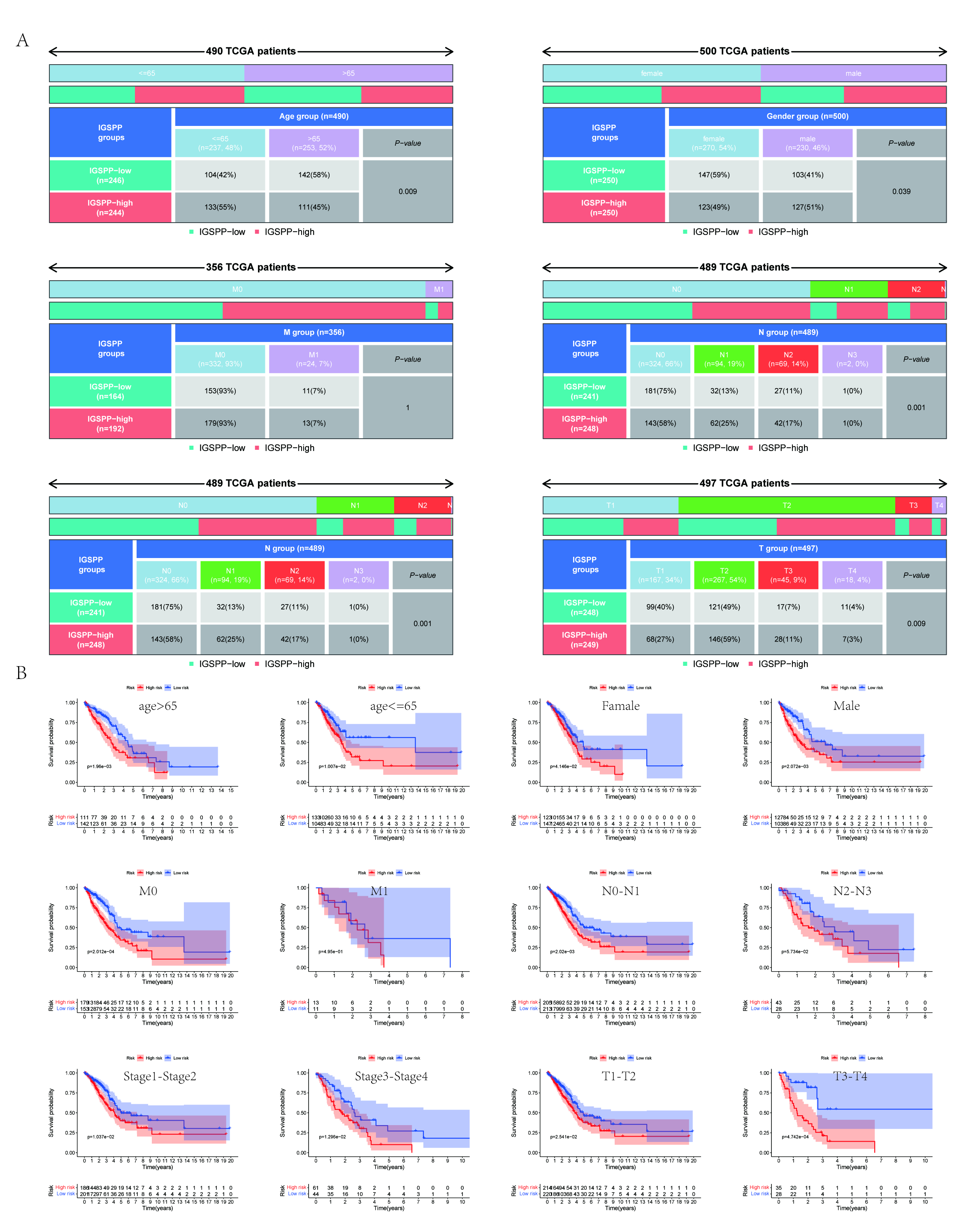

Supplement: Supplementary file 2 — Supplementary Figures. [file 41598_2022_12301_MOESM2_ESM.zip › Supplementary Figures/Figure S5.tif]

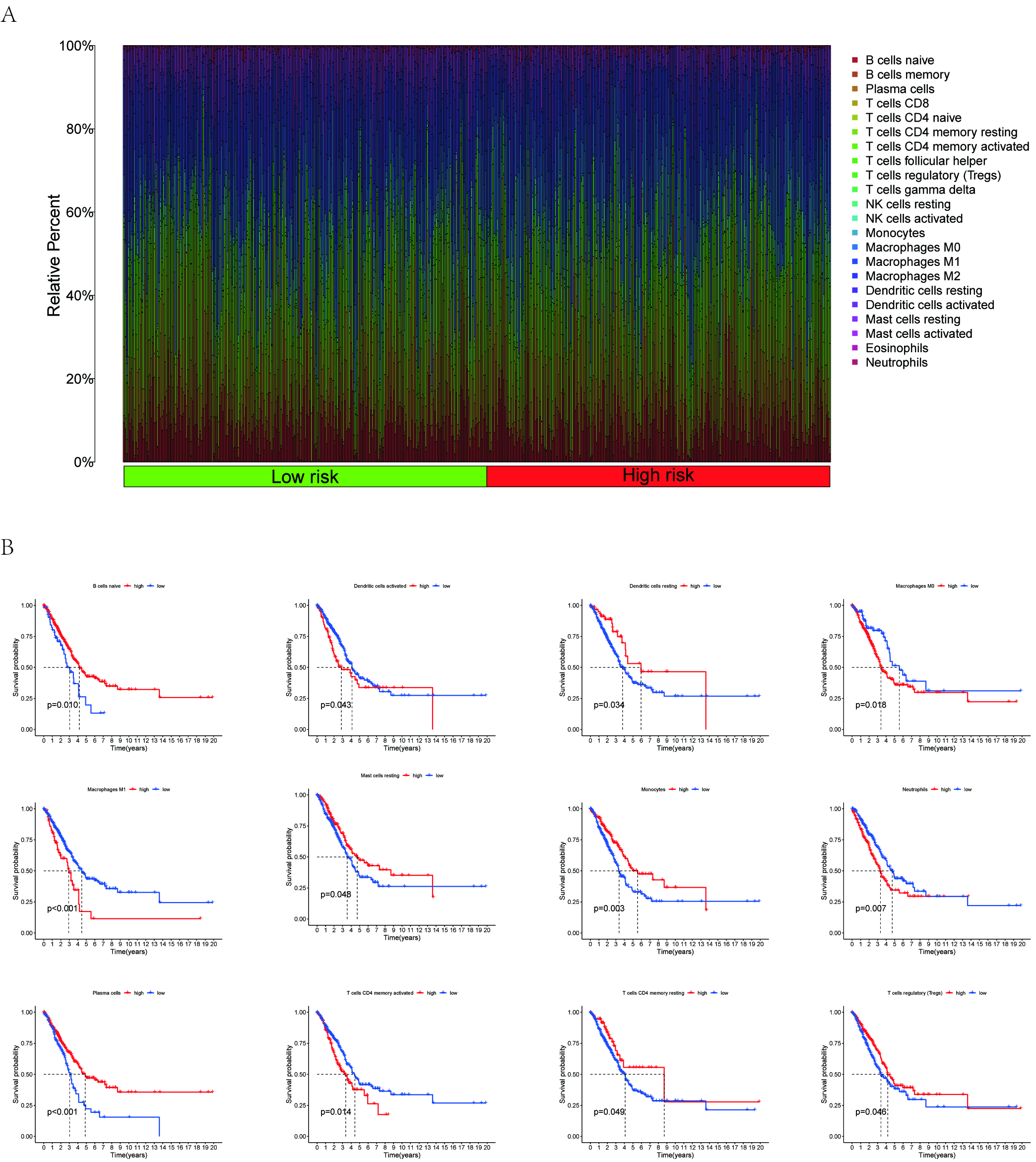

Supplement: Supplementary file 2 — Supplementary Figures. [file 41598_2022_12301_MOESM2_ESM.zip › Supplementary Figures/Figure S6.tif]

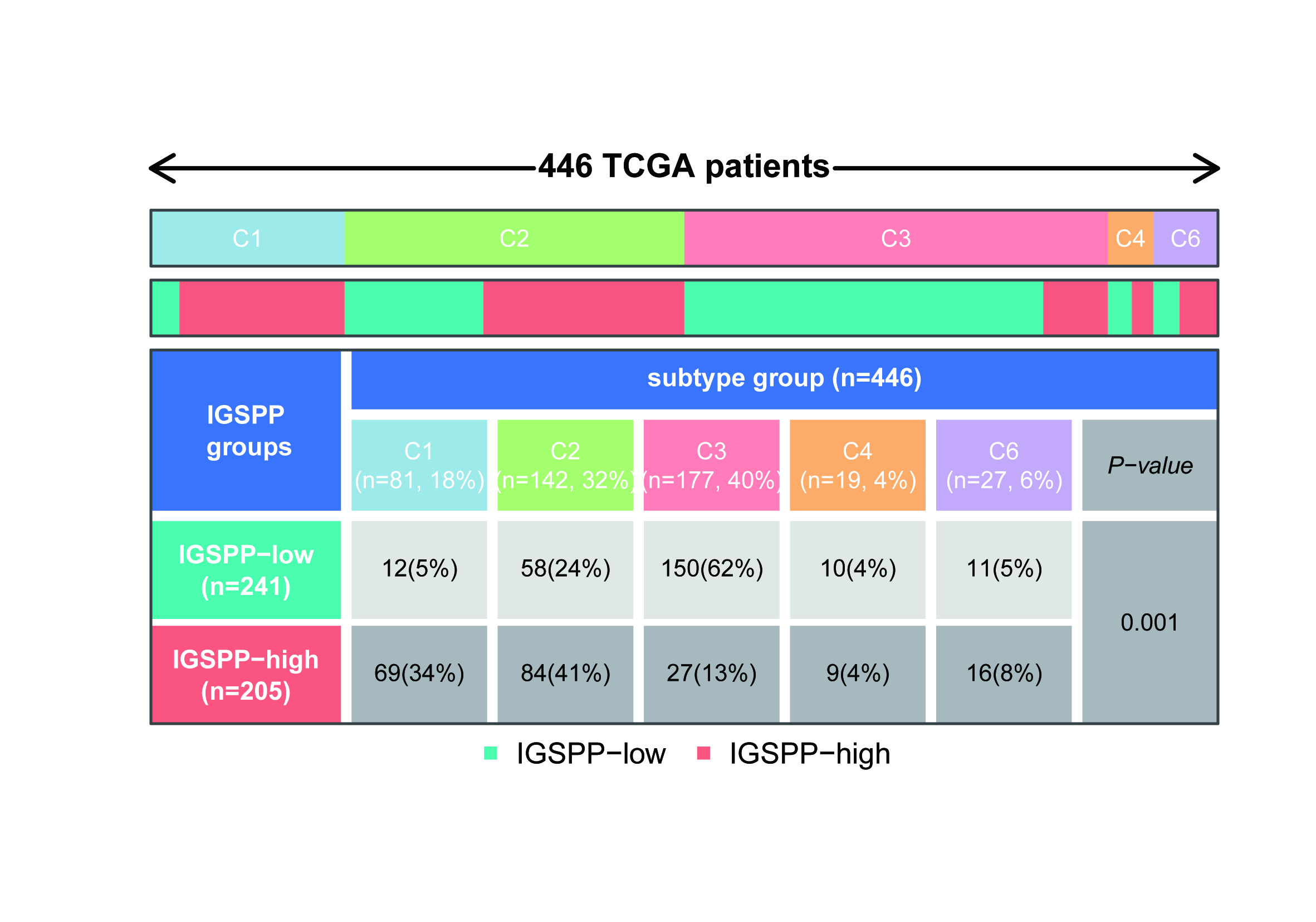

Supplement: Supplementary file 2 — Supplementary Figures. [file 41598_2022_12301_MOESM2_ESM.zip › Supplementary Figures/Figure S7.tif]
